# Can you help me? Using others to offload cognition

**DOI:** 10.3758/s13421-024-01621-9

**Published:** 2024-08-22

**Authors:** Kristy L. Armitage, Jonathan Redshaw

**Affiliations:** School of Psychology, The University of Queensland, Level 3, McElwain Building (24A), St Lucia, QLD 4072 Australia

**Keywords:** Memory, Problem solving, Cognitive offloading, Social cognition, Metacognition

## Abstract

One of the most ancient and widely used forms of cognitive offloading is the outsourcing of cognitive operations onto other humans. Here, we explore whether humans preferentially seek out and use information from more competent compared with less competent others in an ongoing cognitive task. Participants (*N* = 120) completed a novel computerised visuospatial working memory task where each trial required them to remember either one, five, or ten target locations and recall them after a brief delay. Next, participants watched two virtual people compete in a distinct memory game, where one performed relatively well, demonstrating a stronger memory, and the other performed relatively poorly, demonstrating a weaker memory. Finally, participants completed the initial memory task again, but this time, either the strong-memory person or the weak-memory person was available to help with recall on each trial. Our results showed that, through observation and without direct instruction, participants acquired beliefs about the virtual people’s cognitive proficiencies and could readily draw upon these beliefs to inform offloading decisions. Participants were typically more likely to ask for help from the strong-memory person, and this tendency was independent from other factors known to drive cognitive offloading more generally, like task difficulty, unaided cognitive ability, and metacognitive confidence.

**Supplementary Information:**

The online version contains supplementary material available at 10.3758/s13421-024-01621-9.

## Introduction

With the digital revolution has come an abundance of opportunities to outsource thought, alongside concerns about having perpetual access to devices that can compensate for our cognitive shortcomings. We are constantly interfacing with our smartphones, computers, or other devices to store information, perform arithmetic, search Google, and navigate our way home—with no good sense of the downstream effects on our unaided cognition. But such behaviours are not entirely unprecedented. Indeed, one of our most pervasive and accessible external ‘thinking tools’ (Weis & Wiese, 2019a) has been around for untold generations: human problem-solvers can outsource our cognitive operations onto other humans. We often rely on others to remind us to carry out delayed intentions, to contribute to brainstorming when faced with novel challenges, or to share competence that differs from our own. We divide and delegate complex tasks and even create transactive memory systems (TMSs) by distributing information across the minds of other group members (Wegner, 1987). Here, we draw attention to the perspective of other humans as prolific and diverse thinking tools and empirically investigate the factors driving our decisions to offload cognitive demand onto them.

To offload cognition means to engage in behaviours that alter the information processing requirements of a task, resulting in a decrease in cognitive demand (Risko & Gilbert, 2016). Such behaviours supplement and extend the potential of unaided cognitive processes, allowing humans to accomplish cognitive feats that may otherwise be impossible. Exploring how people effectively manipulate their bodies or objects in the physical environment to achieve such feats has been the predominant focus of the cognitive offloading literature to date (e.g., Armitage et al., 2020; Gilbert et al., 2020; Meyerhoff et al., 2021; Risko & Dunn, 2015; Risko et al., 2014; Scarampi & Gilbert, 2021; Weis & Wiese, 2019a, 2019b). Across such studies, the internal demand of a cognitive task has been widely recognised as a critical factor in the decision to offload, such that increases in objective task difficulty are often matched with increases in the frequency of cognitive offloading (Dunn & Risko, 2015; Gilbert, 2015a; Risko & Gilbert, 2016). Accordingly, people will typically offload more frequently if their unaided abilities are relatively poor and less frequently if their unaided abilities are relatively strong (Gilbert, 2015b).

Decisions to offload appear to be mediated by metacognitive beliefs about the difficulty of a task and the level of competency required to carry out the task unaided (Boldt & Gilbert, 2019; Hu et al., 2019; Risko & Gilbert, 2016). Several studies have found that lower confidence in unaided cognitive abilities leads to higher rates of cognitive offloading (Boldt & Gilbert, 2019; Gilbert, 2015b). Critically, however, these beliefs can be inaccurate, leading to offloading decisions that are not well-calibrated with genuine cognitive demand. For example, in a mental rotation task, participants deployed an ‘external normalisation’ technique (i.e., head tilting) to orient themselves towards rotated stimuli more frequently on trials that they incorrectly believed to be more challenging (Dunn & Risko, 2015). Similarly, in a memory task, participants with lower confidence in their unaided abilities set more reminders to aid future recall, even though these beliefs about their unaided memory proficiency were unrelated to objective performance (Gilbert, 2015b). Offloading decisions can also be affected by preconceived beliefs about the utility of a particular object as a cognitive aid. For example, one recent study found that if participants were falsely led to believe that an available aid was unreliable, then their likelihood of offloading substantially decreased, regardless of its actual reliability (Weis & Wiese, 2019a).

### Social offloading

Social offloading is defined as “any shared task-based situation in which an individual is able to leverage agents in the social world (either implicitly or explicitly) to facilitate their own cognitive performance” (Tufft & Richardson, 2020). In some cases, social offloading may be used to free up cognitive resources through sharing demands with other agents with task-relevant expertise (Weis & Wiese, 2020). In TMSs, for example, different knowledge and skills are possessed by each member of a group, creating a complex memory system that exceeds the capacity of any singular individual within the system (Risko & Gilbert, 2016; Wegner, 1987). A successful TMS requires each member to possess an understanding of other members’ relevant knowledge or capabilities. Such expertise is therefore often explicitly delineated, such as in workplaces where position titles and seniority hierarchies are officially recognised (Peltokorpi, 2008). It remains uncertain, however, which mechanisms underpin our decisions to outsource cognitive operations onto other humans and whether we preferentially seek help from more competent others in contexts where expertise must be inferred from relevant actions.

Memory is a particularly suitable domain for exploring the factors that drive social offloading, given that forgetting is a ubiquitous experience for which people often seek external support. One relevant line of research has focused on how social factors influence memory performance using a variations of an explicit memory cueing (EMC) paradigm (see O’Connor et al. 2010), where participants study a list of words before completing a recognition test where they occasionally receive cues that an item is ‘likely old’ or ‘likely new’. Participants indicate their own recognition responses using a confidence rating scale, which ranges from ‘very confident old’ to ‘very confident new’. In Jaeger et al. (2012), participants were falsely led to believe that the cues were provided by two previous participants. One fictive participant was a mostly valid source of information, providing accurate cues on 75% of trials, and the other was a less valid source of information, providing accurate cues on either 50% of trials (Experiment 1) or 25% of trials (Experiment 2), and importantly, the actual participants were not explicitly informed about the validity of information provided by each fictive participant. Experiment 1 showed that participants were largely insensitive to the differences in the validity of the two sources, similarly conforming to both more valid and less valid cues. In Experiment 2, participants were more strongly influenced by the more valid compared with the less valid source (see Krogulska et al., 2023, Experiment, 2 for a similar effect), but nonetheless continued to treat information from both sources as generally valid. More recent work has indicated that participants become more sensitive to differences in source validity when presented with a pretest training phase that provides explicit feedback about recognition decisions (Zawadzka et al., 2016, Experiment 3), but not when presented with a pretest training phase that involves observing both sources in a different but relevant context (Krogulska et al., 2023, Experiment 1).

In typical EMC tasks, however, exposure to memory cues provided during the recognition test are unavoidable for participants (and thus provide a measure of whether participants conform to the information provided rather than whether they actively seek out such information). Indeed, recent research has indicated that information presented in such a manner is difficult to ignore, even if participants are instructed to do so (Selmeczy & Dobbins, 2017). To answer the question of *when* and *how* individuals use external social memory cues, Koop et al. (2021) adapted the EMC paradigm to explore how participants ‘seek out’ this information when desired, by initially hiding cues from participants. As in Jaeger et al. (2012), participants were not explicitly informed about the validity of cues provided from each social source. Across two experiments, participants sought out cues more frequently under conditions of uncertainty and were substantially influenced by the content of those cues, agreeing with the presented information over 80% of the time. However, participants did not preferentially seek information from more valid compared with less valid sources, and there was inadequate statistical power to examine whether the more valid source had a stronger impact on participant responses than the less valid source.

### The current study

The current literature indicates that when we are selecting between multiple human helpers with varying levels of competence in the same cognitive domain, our help-seeking preferences may not follow any discernible patterns if expertise is not explicitly delineated (Koop et al., 2021). Here, we implement a novel task to explore whether participants can infer other people’s cognitive competencies by observing their performance in a different, but relevant context, and use these inferences to inform their social offloading decisions. Our novel task allows for (i) a uniquely sensitive measure of cognitive offloading, where participants can offload a *proportion* of overall memory demand onto other people, (ii) the ability to directly manipulate task difficulty, which has not been incorporated in previous work, and (iii) an avenue for measuring the discrete influences of changes in task difficulty, unaided competence, and metacognition on social offloading decisions.

Participants completed a novel computerised visuospatial working memory task, where each trial required them to remember either 1, 5, or 10 target locations and recall them after a 5-second delay. Before beginning the task, participants made predictions about their unaided performance at each difficulty level, and then completed the task without the option to offload memory demands (phase 1). Participants then watched two virtual people compete in a different visuospatial memory game, where one person successfully recalled the majority of items (i.e., the strong-memory person) and the other recalled few items (i.e., the weak-memory person). Indeed, this is akin to how we often form judgements of others’ cognitive expertise in real life—by witnessing them outperform others, whether it be through receiving competitive school or university grades, notable awards, or job promotions. Finally, participants completed the computerised memory task again, but this time, either the strong-memory person or the weak-memory person was available to help with recall on each trial (phase 2).

In our preregistration (https://osf.io/teq2x/), we hypothesised that participants would offload memory demand onto the virtual people more frequently (i) on difficult trials compared with easy trials (Gilbert, 2015a), (ii) if their actual unaided abilities were relatively poor (Gilbert, 2015b), and (iii) on trials where they believed they would perform poorly (Boldt & Gilbert, 2019; Gilbert, 2015b). We also predicted that participants would preferentially ask for help from the person that ostensibly had the most relevant competence for the task, such that they would offload more frequently onto the strong-memory helper than the weak-memory helper. However, we did not make any specific predictions about how each helper’s cognitive competence may interact with these other factors.

Our experimental design also permitted us to explore several other theoretically interesting questions about the factors affecting social offloading, which were preregistered with no specific hypotheses formed prior to data collection. For instance, we explored whether the above factors influenced not only whether participants chose to offload at all but also their threshold for offloading (i.e., the point at which they chose to offload during a trial). We also explored whether participants’ offloading decisions were driven by their ongoing unaided accuracy on the relevant trial, and whether there were differences in unaided memory performance across phases.

## Method

### Participants

An a priori power analysis indicated that for assumed small-to-medium effect sizes (equivalent to *d* = 0.3), a sample of 119 participants was required to have a 90% chance of detecting a significant effect (α = 0.05, two-tailed). As preregistered on the Open Science Framework (https://osf.io/teq2x/), we therefore recruited 120 Australian residents (60 men, 60 women), with the final sample aged between 17 and 69 years (*M* = 23.56, *SD* = 5.66) and having lived in Australia between 0 and 69 years (*M* = 9.90, *SD* = 10.37). Recruitment was conducted through SONA (a research management system), word-of-mouth, and social media. An additional nine participants were excluded due to technological issues (*n* = 7), failing the primary manipulation check (see ‘Manipulation checks and debriefing’ section, *n* = 1), and attending a session after the preregistered sample was complete (*n* = 1). All participants had normal or corrected-to-normal vision and hearing, and did not have any neurological, psychotic, or mood disorders. Participation was voluntary, and participants received $20 AUD for completing the 40-minute session (one additional participant was recruited without a payment incentive and was excluded from analyses). The study was approved by a Human Research Ethics Committee.

### Testing session outline

#### Consent procedures and framing of the study

To meet the aims of the study, it was important to maximise the extent to which participants treated the virtual people (i.e., the strong-memory and weak-memory helpers) as real people. As approved by the ethics review board, we therefore did not explicitly tell participants that the virtual people were simply programmed to respond in a particular way until after the participants had completed the study. Before signing the consent form, all participants were presented with a written overview of the task that highlighted that some information about the study would be provided only after their participation was complete.

#### Task instructions

Participants completed a 30-minute computerised memory game, which was programmed in Python using PsychoPy Version 2020 1.3 (Peirce et al., 2022) and has been made publicly available on the Open Science Framework (https://osf.io/teq2x/). The paradigm was inspired by a more basic memory offloading task designed by Bulley et al. (2020), in which children could set reminders to help themselves recall the location of hidden targets after a delay. In our task, all written instructions were presented on-screen, accompanied by visual demonstrations, and participants could read at their own pace, using the space bar to progress to the next instruction (see S1 in the supplementary materials for full instructions).

**Fig. 1:**
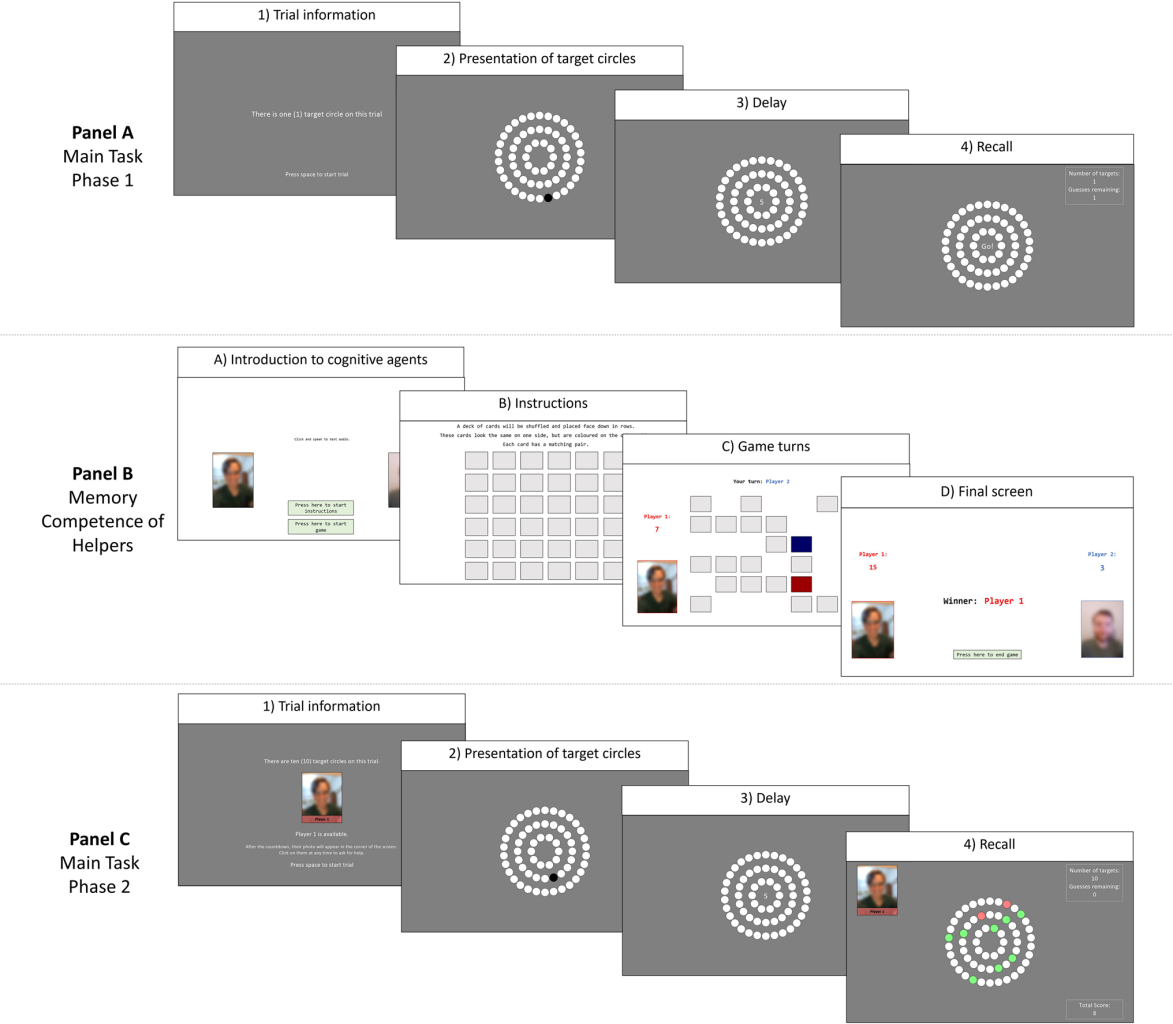
**Panel A** shows an illustration of phase 1 of the computerised memory task. On each trial, participants first received information about the number of targets (either 1, 5, or 10) in the upcoming trial (Box 1), then watched the presentation of target circles one at a time (Box 2) followed by a 5-s delay (Box 3), before attempting to recall the target circles (Box 4). **Panel B** shows an illustration of the video demonstration, in which participants watched two virtual people compete in a computerised game of Concentration (also known as Memory). **Panel C** shows an illustration of phase 2 of the computerised memory task. This was the same as phase 1, except participants had the opportunity to ask the available person (either the strong-memory or weak-memory helper) for help with recalling target circles (green = correct; red = incorrect). The photos of the virtual helpers have been blurred for anonymity as they were provided by real people. All instructions can be found in S1 in the supplementary materials, and a complete version of the task can be found online at https://osf.io/teq2x/ (Colour figure online)

The memory game required participants to memorise and recall the location of ‘target circles’ among an array of 60 white identical circles arranged in three concentric rings (see Fig. 1, Panel A). At the beginning of a trial, either 1, 5, or 10 target circles would consecutively appear on screen, turning black for 0.75 seconds before returning to white, such that each one returned to white before the presentation of the next target circle. Each circle could only present as a target once per trial. For each trial, a random number generator was used to select the target circles, with the constraint that targets would not be adjacent, and had to be spread relatively evenly across the inner, middle, and outer rings (see S2 in the supplementary materials for complete counterbalancing information). A 5-second countdown commenced immediately after the presentation of the final target circle, after which participants used the mouse to click on the circles they believed to be targets. During the initial instruction phase, participants practiced clicking on circles, and received immediate feedback after each click, such that selected target circles turned green and selected nontarget circles turned red (see Fig. 1, Panel B). They were also informed that they would only be allowed the same number of guesses (clicks) as the number of target circles on the trial.

#### Predicted accuracy measures

Next, participants made predictions about their recall performance on trials of each difficulty level (1 target, 5 targets, and 10 targets). For each difficulty, participants were shown an example of the trial, including the 5-second delay, and were then asked to report the number of target circles they believed they would accurately recall on a scale from none (0) to all (total number of targets) in increments of 0.1. Participants’ ratings were divided by the highest possible rating on the relevant scale to produce a *predicted accuracy* variable with an equivalent range for each difficulty level (0 to 1).

#### Main task phase 1 (unaided memory)

Participants then completed nine task trials, made up of three 1-target trials, three 5-target trials, and three 10-target trials (see Fig. 1, Panel A). The trials were organised into three ‘blocks’ of three trials, with each block including one trial of each difficulty in different orders. Across the first three trials (block 1), participants experienced the task in increasing difficulty, starting with a 1-target trial, followed by a 5-target trial, followed by a 10-target trial. The remaining phase 1 trials were presented in a way that counterbalanced the overall order of the trials across each difficulty level (see S2 in the supplementary materials for complete counterbalancing information). As in the instruction phase, participants received immediate feedback as to whether they selected a target or nontarget circle during each trial. For each trial, the number of correctly recalled target circles was recorded and divided by the total number of target circles to produce a *phase 1 accuracy* variable with an equivalent range for each difficulty level (0 to 1).

#### Memory competence of helpers

At the end of phase 1, participants watched a prerecorded video of two people competing in a computerised game of Concentration (also known as Memory). The video included photos and voice recordings voluntarily provided by real people, and participants were not informed that it was prerecorded, to increase the extent to which they felt like they were viewing a genuine attempt at the game. In the beginning, 36 identical grey rectangular cards were arranged in a 6 × 6 grid (see Fig. 1, Panel B). The players, referred to as Player 1 and Player 2, took turns selecting pairs of cards. If the colour on the underside of the cards matched, both cards were removed and the player’s score increased by 1, but if they did not match, both cards were returned facedown. To ensure the game felt as realistic as possible, the players typically chose the second card in the pair quickly if they had prior information about its location (for example, if their first card was orange, and the location of the second orange card had been revealed in a previous turn), and more slowly if they were simply guessing.

At the end of the game, one player had secured 15 of the 18 possible pairs (83.33% successful) and the other had secured three pairs (16.67% successful). Although one player clearly outperformed the other, it was never explicitly stated that they had the better memory, allowing participants to form their own beliefs about each player’s memory competence through observation (Norman, 2014). Four versions of this prerecorded video were created for counterbalancing purposes, where the sex of the players (either both women or both men) and the winning player (either Player 1 or Player 2) were varied, and we ensured that an equal number of men and women participants saw each version.

#### Main task phase 2 (aided memory)

Next, participants completed the initial memory task again, but this time, they could choose to ask the two players that had competed in the Concentration game to help recall target circles. On each trial, either Player 1 or Player 2 was available to help, and their picture would appear in the corner of the screen. Participants could click on this picture at any point during the trial, and the available helper would then complete all remaining guesses. These guesses appeared on the screen consecutively, as if a real person were clicking the circles one at a time, but critically, the accuracy of these selections varied according to the memory competence of the available helper. Both helpers were programmed to demonstrate an equivalent memory proficiency as in the prerecorded memory video, such that each time they selected a circle, the strong-memory helper had an 83.33% chance of correctly selecting a target circle (matching 15/18 correct card pair selections in the video), and the weak-memory helper had a 16.67% chance of correctly selecting a target circle (matching 3/18 correct card pair selections in the video). This phase included six trials of each difficulty level, where half of the trials had the strong-memory helper available, and the other half had the weak-memory helper available (18 trials total). The order of trials was determined according to the following constraints, and then reversed to create another order: (i) trials with the same combination of difficulty and available helper were separated by at least two other types of trials, and (ii) the same helper could only appear on a maximum of two consecutive trials (see S2 in the supplementary materials for complete counterbalancing information).

One consequence of allowing participants to immediately ask for help and avoid relying on their unaided abilities is that it increases the likelihood of them offloading merely to avoid effort (Sachdeva & Gilbert, 2020). To mitigate this issue, participants’ total scores were displayed on-screen throughout phase 2, and increased each time a target circle was correctly recalled by either themselves or their selected helpers. Importantly, participants were also made aware that five financial rewards (ranging from $50 AUD to $150 AUD) were available for those with the highest total scores at the end of data collection.

On each trial, we recorded whether the participant asked for help at any point (*binary offloading* variable). We also recorded their threshold for asking for help, which was calculated by dividing the number of guesses made before clicking the helper by the number of guesses permitted for the relevant trial (*offloading threshold* variable, with participants that did not ask for help assigned the maximum threshold score of 1). A *phase 2 unaided accuracy* variable was recorded as the proportion of correct guesses made before asking for help (e.g., if a participant made two correct guesses and no incorrect guesses before offloading on a 10-target trial, their score on this variable would be 1). Note that this calculation differs from *phase 1 accuracy* variable, which represented the proportion of correct guesses made out of the total number of guesses available (e.g., a participant would have to make 10 correct guesses and no incorrect guesses on a 10-target trial to achieve a score of 1). We also calculated a combined proportional accuracy score of the participant and helper (*phase 2 combined accuracy* variable).

#### Manipulation checks and debriefing

After the main task, participants completed six manipulation checks. First, they were asked to recall which helper won the memory game with coloured cards, and any participant that answered incorrectly was excluded from the final dataset. Participants were then asked to rate each helper’s memory ability on a 100-point scale from ‘extremely bad’ (0) to ‘extremely good’ (100). In the next two questions, they were asked to rate how happy and surprised they believed each helper was when they correctly recalled a target circle on a 100-point scale from ‘not happy/surprised at all’ (0) to ‘extremely happy/surprised’ (100). For these scales, significant differences between the helpers would imply that participants attributed at least some element of humanness to them, such that they expected the helpers to have different emotional and cognitive experiences in response to their performance on the task. Participants were then asked to report the total score on a hypothetical trial, where seven target circles were successfully recalled between the participant and available helper, to ensure they understood that it was both their own success and the success of the helpers that contributed to their total score. Finally, participants were directly asked to rate the extent to which it felt like each helper was a real person, on a 100-point scale from ‘not real at all’ (0) to ‘extremely real’ (100).

The experimenter provided all participants with a full verbal and written debrief, which included explaining that the virtual helpers were not real people, and participants were given the opportunity to ask any questions before being paid and thanked for their time. At the conclusion of data collection for all participants, the top five scoring participants were contacted to receive their financial prize.

## Results

### Analysis strategy

**Fig. 2:**
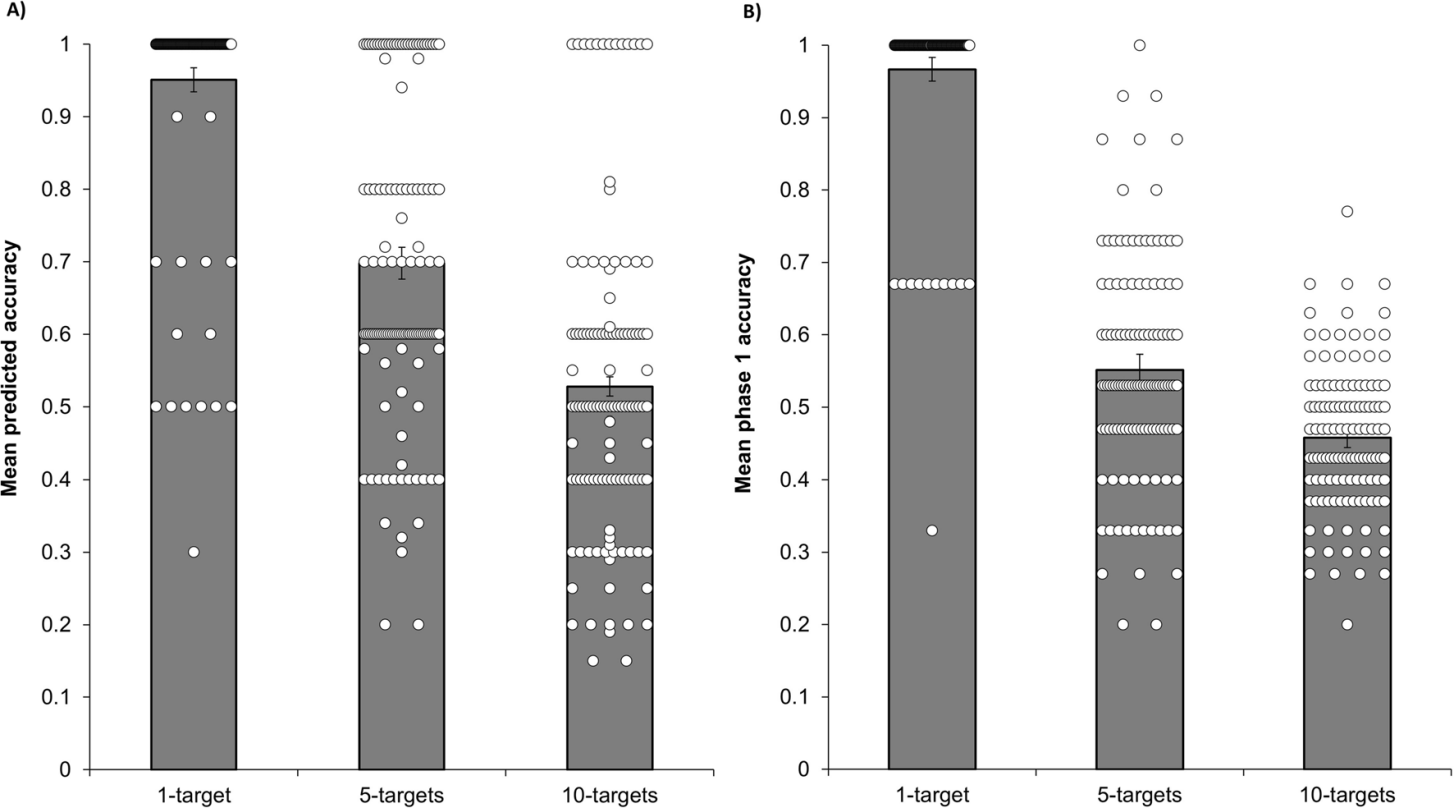
A visual representation of participants’ predictions made about their own future performance (mean *predicted accuracy*; **Panel A**) and their actual unaided performance in phase 1 (mean *phase 1 accuracy*; **Panel B**), as represented by the grey bars. The white dots represent individual participant data points. Error bars represent standard errors

**Fig. 3:**
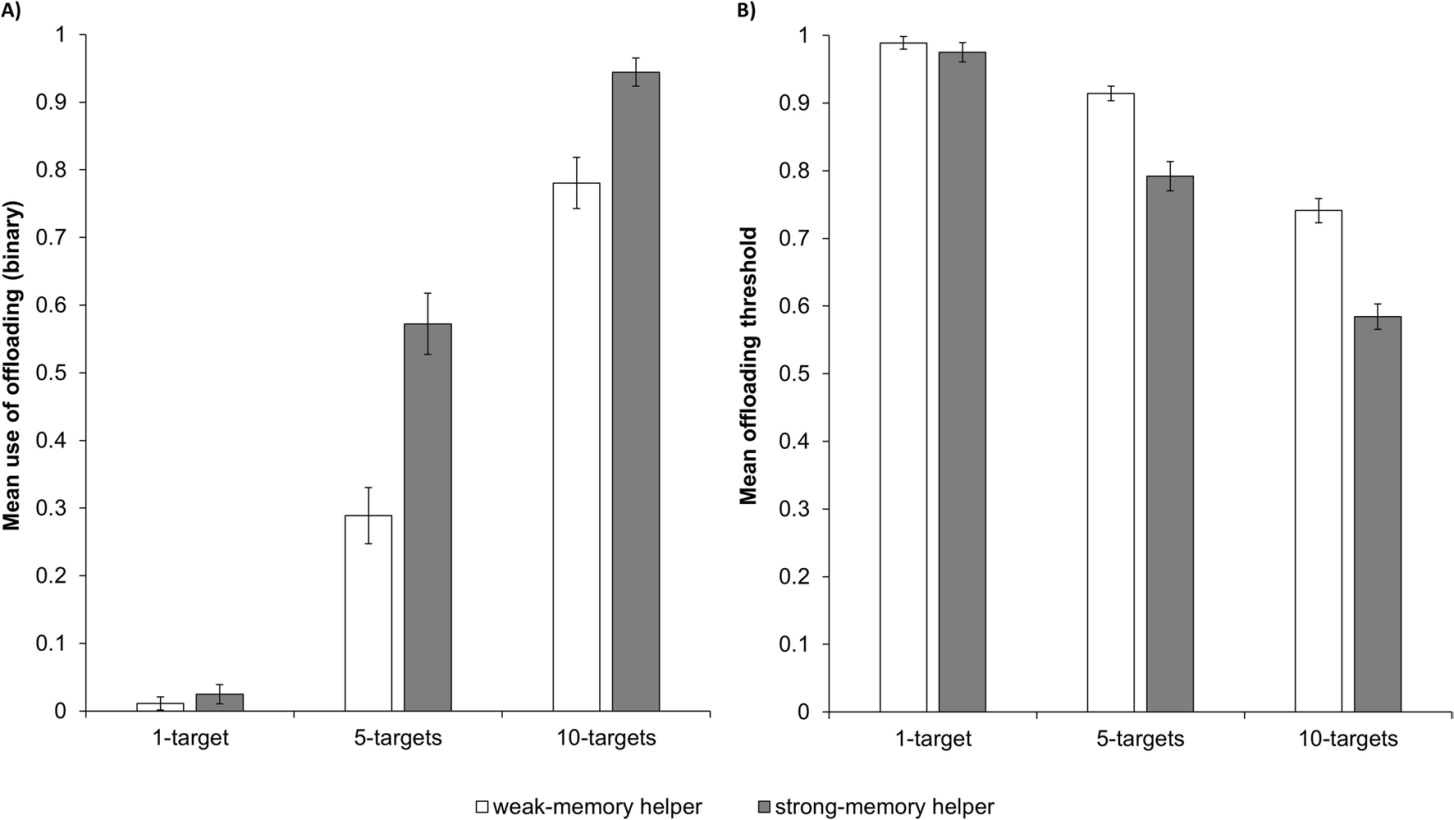
Mean use of offloading (binary variable; **Panel A**) and mean offloading threshold (e.g., the point at which participants chose to offload during a trial; **Panel B**) at each difficulty level, where the white bars represent trials where the weak-memory helper was available and the grey bars represent trials where the strong-memory helper was available. Error bars represent standard errors

**Fig. 4:**
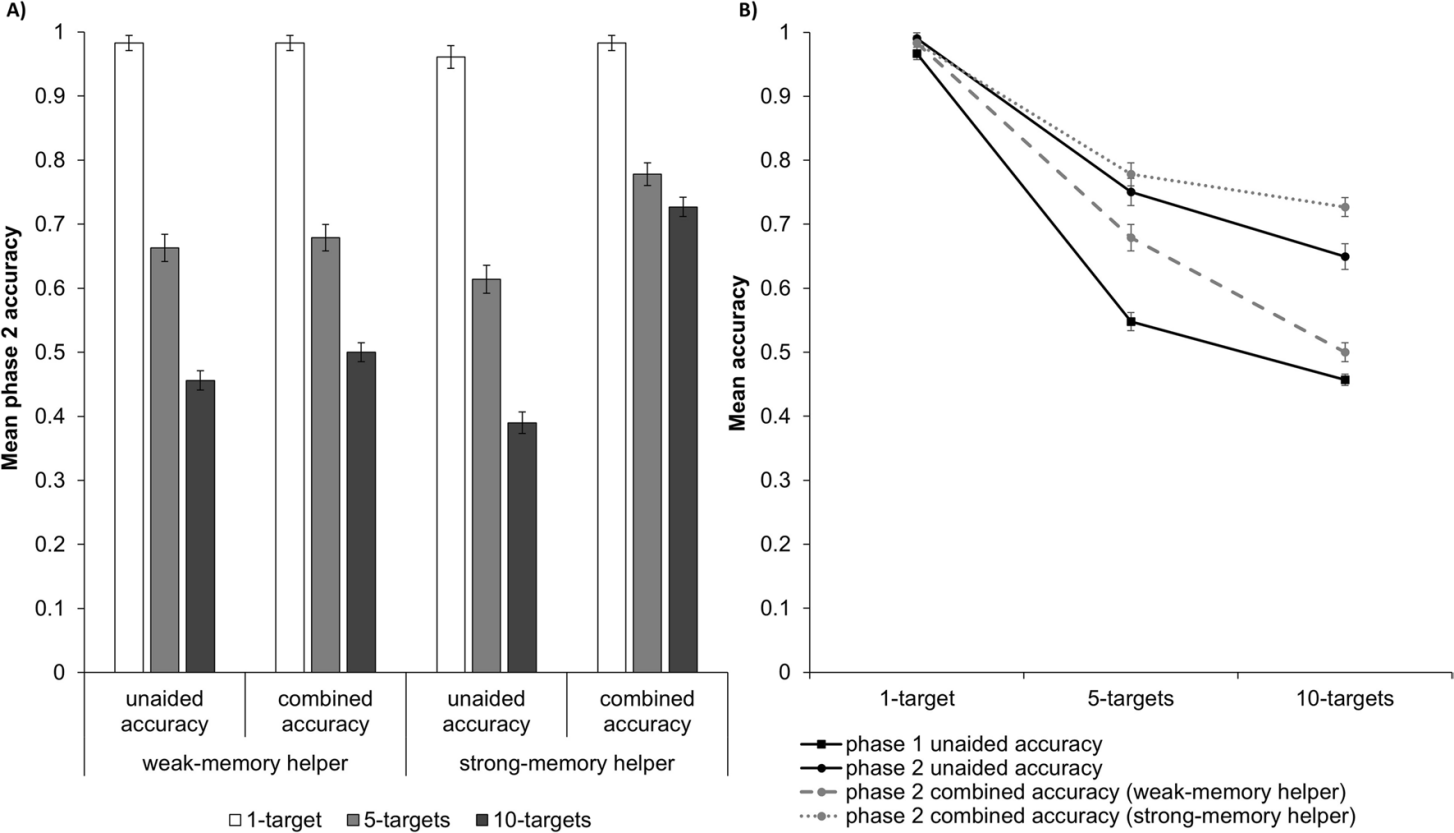
**Panel A** compares participants’ mean unaided accuracy (the proportion of target circles correctly recalled by the participant prior to offloading) and combined accuracy (the proportion of target circles correctly recalled by either the participant or helper by the end of the trial) across each difficulty level (1, 5, and 10 targets) in phase 2. The left-hand side of the graph shows accuracy for trials on which the weak-memory helper was shown, and the right-hand side of the graph shows accuracy for trials on which the strong-memory helper was shown. Error bars represent standard error. **Panel B** shows the mean unaided accuracy across both phases for each difficulty level. The black line with the square marker shows the mean unaided accuracy in phase 1, and the black line with the circle marker shows the mean unaided accuracy in phase 2 (i.e., before any offloading decision). The grey lines indicate the mean combined accuracy in phase 2 for each difficulty level, for trials with the strong-memory helper (dotted grey line) and weak-memory helper (dashed grey line). Error bars represent standard error

Data from both phases were analysed using generalised linear mixed models (GLMMs) with a random intercept for each participant, using the statistical program SAS 9.4 (SAS Institute Inc., 2013). All binary dependent variables were modelled using a binomial distribution and a logit link function. Bonferroni corrections were applied to *p* values for all follow-up tests involving multiple comparisons. All preregistered analyses were run and are reported below, with some adjustments. First, one-target trials were removed from all analyses after observing participants performing at ceiling on these trials, showing minimal variability in either their accuracy or offloading decisions (see Figs. 2, 3, and 4). Second, given the wide age range of our final sample, we decided to control for age in every model. Third, for each analysis, we ran a base model including all relevant main effects (rather than progressively adding them, as preregistered). We then compared each interaction model, which included all main effects and the relevant interaction term, with the base model using a likelihood ratio test. Full model details for all analyses (including likelihood ratio tests and directional information for all effects) can be found in the supplementary materials (see S3 to S10). Preregistered focal analyses, preregistered exploratory analyses, and exploratory analyses that were not preregistered have been clearly labelled throughout.

### Predicted accuracy measures

A nonparametric Friedman’s test showed a statistically significant difference between predicted accuracy for 1-target, 5-target, and 10-target trials, χ^2^(2) = 174.61, *p* < .001. Post hoc Wilcoxon signed-rank tests revealed that the predicted accuracy for 1-target trials (*M* = 0.95, *SD* = 0.14) was significantly higher than for 5-target trials (*M* = 0.70, *SD* = 0.22), *z* = −7.99, *p* < .001, which in turn was significantly higher than for 10-target trials (*M* = 0.53, *SD* = 0.22), *z* = −7.94, *p* < .001 (see Fig. 2, Panel A).

### Phase 1

#### Accuracy (preregistered focal analyses)

Phase 1 accuracy was modelled as a function of task difficulty (5 targets or 10 targets), predicted accuracy (continuous, mean-centred), and trial (the trial number for the specific type of trial, 1–3), controlling for age (continuous). Significant main effects of task difficulty and trial indicated that higher accuracy was recorded on (i) 5-target (*M* = 0.55, *SD* = 0.24) compared with 10-target trials (*M* = 0.46, *SD* = 0.14)*, F*(1, 597) = 48.93, *p* < .001, *η*_*p*_^2^ = .08 (*b = −*0.09; see Fig. 2, Panel B), and (ii) on later trials compared with earlier trials, *F*(1, 597) = 4.51, *p* = .034, *η*_*p*_^2^ = .01 (*b* = 0.02). The main effect of predicted accuracy was nonsignificant, *F*(1, 597) = 1.54, *p* = .215, *η*_*p*_^2^ < .01 (*b* = −0.05), and post hoc paired-samples *t* tests revealed that participants significantly overestimated how well they would perform on both 5-target, *t*(719) = 15.00, *p* < .001, and 10-target trials, *t*(719) = 7.00, *p* < .001. The interaction between task difficulty and predicted accuracy was also nonsignificant, *F*(1, 596) = 0.89, *p* = .345, *η*_*p*_^2^ < .01.

### Phase 2

#### Binary offloading decisions (preregistered focal analyses)

The binary offloading variable (yes or no) was modelled as a function of task difficulty, helper competence (strong memory or weak memory), trial, phase 1 accuracy (proportion of correct phase 1 responses for each difficulty level), and predicted accuracy, controlling for age (base model). Significant main effects of task difficulty, helper competence, and phase 1 accuracy indicated higher rates of offloading (i) on 10-target (*M* = 0.86, *SD* = 0.35) compared with 5-target trials (*M* = 0.43, *SD* = 0.50), *χ*^2^(1, 1315) = 205.15, *p* < .001, *w* = 1.31 (*b* = 2.55; see Fig. 3, Panel A; Cohen, 1992), and (ii) when the available helper had a stronger (*M* = 0.76, *SD* = 0.43) compared with weaker (*M* = 0.53, *SD* = 0.50) memory, *χ*^2^(1, 1315) = 104.61, *p* < .001, *w* = 0.93 (*b* = 1.56), and (iii) on trials where participants had performed poorly in phase 1, *χ*^2^(1, 1315) = 11.84, *p* = .001, *w* = 0.31 (*b* = −2.47). The main effect of phase 1 accuracy significantly interacted with task difficulty, *χ*^2^(1, 1314) = 4.00, *p* = .046, *w* = 0.18, indicating that the effect was more pronounced on 10-target trials, *χ*^2^(1, 1314) = 11.88, *p* = .001, *w* = 0.31, compared with 5-target trials, *χ*^2^(1, 1314) = 6.18, *p* = .026, *w* = 0.23. All other main effects and interactions were nonsignificant, including all interactions involving helper competence (see S4 in the supplementary materials).

##### Sex effects

A preregistered exploratory analysis revealed that the interaction between task difficulty and phase 1 accuracy significantly varied by participant sex, *χ*^2^(2, 1312) = 6.63, *p* = .036, *w* = 0.24. Follow-up analyses revealed that for women participants, lower phase 1 accuracy was associated with more frequent offloading on both the 5-target, *χ*^2^(1, 1312) = 12.00, *p* = .001, *w* = 0.45, and 10-target trials, *χ*^2^(1, 1312) = 19.50, *p* < .001, *w* = 0.57. For men participants, however, lower phase 1 accuracy was not associated with offloading on either type of trial in phase 2, *χ*^2^s < 6.21, *p *values > .051, *w *values < 0.32. Another preregistered exploratory analysis revealed a significant interaction between participant sex and helper sex, *χ*^2^(1, 1317) = 4.64, *p* = .031, *w* = 0.20, such that men participants, *χ*^2^(1, 1317) = 8.74, *p* = .006, *w* = 0.38, but not women participants, *χ*^2^(1, 1317) = 0.01, *p* > .999, *w* = 0.01, offloaded more frequently when the available helpers were women compared with men. No other significant sex effects were detected across all analyses (see S8 in the supplementary materials).

#### Offloading threshold (preregistered exploratory analyses)

The continuous offloading threshold variable was modelled as a function of task difficulty, helper competence, trial, phase 1 accuracy, and predicted accuracy, controlling for age (base model). A significant main effect of difficulty, *F*(1, 1315) = 275.45, *p* < .001, *η*_*p*_^2^ = .17 (*b* = −0.17), revealed that participants had a lower offloading threshold (i.e., offloaded earlier in the trial) on 10-target (*M* = 0.66, *SD* = 0.22) compared with 5-target trials (*M* = 0.85, *SD* = 0.21). Notably, however, the descriptive data for this effect indicate that participants were making a *higher total number* of unaided guesses on 10-target trials (*M* = 6.60 guesses) than was possible on 5-target trials, such that they were not simply offloading after a particular number of searches across all trials (see Fig. 3, Panel B). The main effects of helper competence and trial were also significant, indicating that participants had a lower offloading threshold (i.e., offloaded earlier in the trial) (i) when the available helper had a stronger (*M* = 0.69, *SD* = 0.25) compared with weaker memory (*M* = 0.83, *SD* = 0.20),* F*(1, 1315) = 229.28, *p* < .001, *η*_*p*_^2^ = .19 (*b* = −0.14), and (ii) on later compared with earlier trials, *F*(1, 1315) = 6.51, *p* = .011, *η*_*p*_^2^ < .01 (*b* = −0.01). The effect of phase 1 accuracy, *F*(1, 1315) = 15.58, *p* < .001, *η*_*p*_^2^ = .01 (*b* = 0.20)*,* significantly interacted with task difficulty, *F*(1, 1314) = 15.06, *p* < .001, *η*_*p*_^2^ = .01. Follow-up analyses revealed that participants had a higher offloading threshold (e.g., offloaded later in the trial) on trials of both difficulty levels where they had performed relatively well in phase 1, with the effect being less pronounced on 5-target, *F*(1, 1314) = 5.04, *p* = .050, *η*_*p*_^2^ < .01, compared with 10-target trials, *F*(1, 1314) = 30.39, *p* < .001, *η*_*p*_^2^ = .02. All other main effects and interactions were nonsignificant, including all interactions involving helper competence (see S5 in the supplementary materials).

#### Accuracy (preregistered exploratory analyses)

Figure 4, Panel A compares participants’ average accuracy before (unaided accuracy) and after (combined accuracy) opting for help across phase 2 trials. Unaided accuracy was calculated as the proportion of target circles correctly recalled by the participant prior to offloading, and combined accuracy was calculated as the proportion of target circles correctly recalled by either the participant or helper by the end of the trial. As seen in Fig. 4, Panel A, asking for help from the strong-memory helper, but not the weak-memory helper, resulted in higher levels of accuracy. Note, however, that scores on the combined accuracy variable were dependent on programming specifications, as each helper was programmed to recall target circles at a prespecified probabilistic rate (83.33% for the strong-memory helper and 16.67% for the weak-memory helper). Across all participants, the actual proportion of correct guesses made by each helper was 81.00% for the strong-memory helper and 17.46% for the weak-memory helper. For this reason, we only report analyses using the unaided accuracy variable in text (but see S6 of the supplementary materials for exploratory analyses of the combined accuracy variable). We use proportional unaided accuracy to allow for interpretable comparisons across trials of varying difficulty levels, but we also report the same models using total unaided accuracy in sections S4 to S7 of the supplementary materials.

##### Effect of phase 2 unaided accuracy on offloading

A preregistered exploratory analysis examined whether a participant’s ongoing unaided accuracy on phase 2 trials influenced their offloading decisions. Both offloading (binary) and offloading threshold were modelled as a function of task difficulty, helper competence, trial, and phase 2 unaided accuracy (continuous), controlling for age. The offloading threshold model revealed a significant interaction between phase 2 unaided accuracy and helper competence, *F*(1, 1315) = 8.27, *p* = .004, *η*_*p*_^2^ = .01, such that participants with poorer unaided performance had a higher threshold for offloading when the available helper had a weak memory, *F*(1, 1315) = 12.72, *p* < .001, *η*_*p*_^2^ = .01, but not when they had a strong memory, *F*(1, 1315) = 0.00, *p* > .999, *η*_*p*_^2^ < .01. This interaction was nonsignificant in the offloading (binary) model, *χ*^2^(1, 1315) = 2.07, *p* = .151, *w* = 0.13.

##### Unaided accuracy across phases

An exploratory analysis compared unaided recall performance across phases, after observing higher means for phase 2 unaided accuracy compared with phase 1 accuracy (see Fig. 4, Panel B). Unaided accuracy across both phases was modelled as a function of difficulty, trial, and phase (phase 1 or phase 2), controlling for age. The significant main effect of phase, *F*(1, 2037) = 410.32, *p* < .001, *η*_*p*_^2^ = .17 (*b* = −0.19), did not significantly interact with difficulty, *F*(1, 2036) = 0.12, *p* = .728, *η*_*p*_^2^ < .01, indicating that, on both 5- and 10-target trials, participants were demonstrating significantly better unaided recall in phase 2 (*M* = 0.64, *SD* = 0.24) compared with phase 1 (*M* = 0.55, *SD* = 0.15). We then conducted the same analyses using a total (rather than proportional) accuracy variable, which revealed a nonsignificant main effect of phase, *F*(1, 2037) = 0.56, *p* = .456, *η*_*p*_^2^ < .01 (*b* = −0.04; see S7 in the supplementary materials). In other words, participants were successfully recalling roughly the same *number* of target circles across both phases but were offloading strategically during the phase 2 trials (i.e., after they had exhausted the targets readily available in memory), resulting in a higher *proportion* of correct responses.

### Manipulation checks

Wilcoxon signed-rank tests were used to determine whether there were significant differences in participants’ perceptions of the available helpers, as assessed in the manipulation check questions. The weak-memory helper was rated as having a significantly worse memory, being happier and more surprised after successfully recalling a target circle and feeling more ‘real’ than the strong-memory helper (see Table 1). Almost all participants (95.83%) correctly responded to the question assessing whether they understood that it was both their own success and the success of the helpers that were recruited to help that contributed to their overall score.

**Table 1:** Mean ratings for manipulation-check questions

	Strong-memory helper	Weak-memory helper	Wilcoxon signed-rank
Memory rating	*M* = 84.73, *SD* = 8.76	*M* = 27.33, *SD* = 16.30	*z* = −9.51, *p* < .001^a^
Happiness rating	*M* = 72.83, *SD* = 16.89	*M* = 75.04, *SD* = 24.96	*z* = −2.19, *p* = .029^b^
Surprise rating	*M* = 43.86, *SD* = 21.87	*M* = 75.23, *SD* = 18.45	*z* = −8.19, *p* < .001^b^
Realness rating	*M* = 48.00, *SD* = 26.43	*M* = 58.62, *SD* = 28.63	*z* = −3.83, *p* < .001^b^

^a^ Based on positive ranks

^b^ Based on negative ranks

*Note.* All scales ranged from 0 to 100 in increments of 1

## Discussion

In our everyday lives, we often find ourselves selecting between multiple human helpers with varying levels of competence in the same domain, and yet it remains unclear *who* and *when* we ask for help if each individual’s expertise is not explicitly delineated (Koop et al., 2021). Here, we designed a novel task that allowed us to explore (i) the factors driving social offloading decisions, and (ii) whether people can make inferences about others’ cognitive competencies by observing their performance in a different but relevant context, and use these inferences to inform their social offloading decisions. Our results demonstrated that such inferences strongly influenced social offloading decisions, with participants more frequently and proactively seeking memory assistance from more competent compared with less competent virtual human helpers. These findings complement earlier work showing patterns of preferential conformity to information provided by more valid compared with less valid memory sources (Jaeger et al., 2012, Experiment 2; Krogulska et al., 2023, Experiment 2).

Our findings further demonstrate that some of the factors driving cognitive offloading more generally, like task difficulty and unaided abilities (Dunn & Risko, 2015; Gilbert, 2015a, 2015b; Risko & Gilbert, 2016), are similarly implicated in the decision to offload onto other people. Task difficulty, for example, predicted participants’ offloading decisions, such that they offloaded significantly more frequently (and had a lower offloading threshold) on trials of higher difficulty (as in Gilbert, 2015a). Participants were also more likely to offload (and had a lower offloading threshold) in phase 2 if they had previously performed relatively poorly on similar trials in phase 1, aligning with earlier work indicating that offloading is often used to strategically compensate for cognitive shortcomings (Armitage & Redshaw, 2022; Gilbert, 2015b). Alternatively, one might suspect this effect was driven by a subset of participants who performed poorly on phase 1 due to a lack of interest or motivation and offloaded in phase 2 in pursuit of completing the task as quickly and effortlessly as possible (despite the financial incentive contingent on performance). If participants with lower phase 1 accuracy were indeed adopting an effort-minimisation rather than performance-maximisation approach (see Sachdeva & Gilbert, 2020), however, then there would have been no reason for them to preferentially seek help from the strong-memory compared with the weak-memory helper. Instead, all focal interactions involving helper competence were nonsignificant, such that participants consistently sought help from the strong-memory compared with the weak-memory helper across trials of varying difficulty, independent of their level of unaided cognitive performance.

We also measured participants’ unaided accuracy in phase 2 (i.e., the proportion of targets successfully recalled prior to offloading) and found significantly higher scores in phase 2 compared with phase 1. That is, participants were strategically offloading *after* they had exhausted the targets readily available in memory, and *before* their unaided performance would have likely declined. Further analyses revealed that if participants were making relatively more errors during phase 2 recall, they showed a higher threshold for offloading on trials where the weak-memory helper was available, perhaps because there were more targets still to find when ongoing unaided performance was lower (and the weak-memory helper was unlikely to find them).

The only consistent non-significant predictor of offloading was participants’ metacognitive predictions of their unaided recall accuracy, which is surprising given the well-established relationship between metacognition and cognitive offloading (Boldt & Gilbert, 2019; Hu et al., 2019; Risko & Gilbert, 2016). This was the case even though participants significantly overestimated how well they would perform on both 5-target and 10-target trials, and even though their social offloading decisions were informed by their *actual* phase 1 accuracy (as opposed to their *predicted* phase 1 accuracy). Given that it was a novel task—and participants only saw one example trial of each difficulty level (without the opportunity for recall) before being asked to predict their performance (cf. Gilbert, 2015b)—it is likely that their beliefs about their own abilities were refined through experience, such that the content of those beliefs after the nine phase 1 trials (i.e., before the offloading phase) no longer reflected their accuracy predictions made before phase 1. Indeed, direct task experience may have similarly prompted participants to update their beliefs about the usefulness of the available cognitive helpers, as suggested by significant changes in participants’ offloading tendencies and accuracy scores across phase 2 trials. Future studies may therefore wish to measure beliefs about self-competence and other-competence both before and after (and perhaps during) each task phase.

Intriguingly, exploratory analyses revealed that some of our findings varied according to participant sex. For instance, women participants’ offloading decisions and thresholds in phase 2 were consistently associated with their unaided performance in phase 1, such that lower unaided ability predicted more frequent and earlier offloading, whereas these relationships were absent or less pronounced in men participants. Similar sex differences have been observed in adolescents, with boys showing lower metacognitive accuracy than girls (Weil et al., 2013) and higher levels of confidence despite performing objectively similarly on a visual perception task (Moses-Payne et al., 2020). Furthermore, although women participants were similarly likely to ask for help from women and men helpers, men participants were significantly more likely to ask for help from women than men helpers. One possible explanation is that men trust women strangers more than they trust men strangers. In a study where men and women were presented with pictures of strangers’ faces on a screen, for instance, men rated women strangers as significantly more trustworthy than women rated men strangers, with the lowest trust level being observed in the men–men pairings (Zhao & Zhang, 2016).

At the conclusion of the experiment, participants rated the weak-memory helper as feeling significantly more ‘real’ than the strong-memory helper, perhaps reflecting their more error-prone, human-like performance in the prerecorded memory game (and the circle task itself). Participants also rated the weak-memory helper as being significantly happier and more surprised after successfully recalling a target circle compared with the strong-memory helper. These findings imply that participants attributed at least some level of humanness to the helpers, such that they perceived them to have different emotional and cognitive experiences in response to their task performance. We note, however, that a replication of the current study with in-person confederates, rather than virtual people, may be useful in verifying the ecological validity of our findings.

There are other limitations of this study that also give rise to interesting avenues for future research. First, although there is some evidence that lab-based cognitive offloading tasks scale up to reflect real-world offloading decisions (Scott & Gilbert, 2024), it is likely that in true social contexts there are more mechanisms at play than were accounted for within the current experiment (see Nadler, 1991; Wills & DePaulo, 1991). Indeed, our preferential help-seeking from more competent compared with less competent others may vary according to whether they demonstrate other desirable characteristics, such as patience or kindness. In some circumstances, asking for help may pose a threat to our self-esteem (Bamberger, 2009; Ryan & Pintrich, 1997), elicit feelings of inadequacy or inferiority (Sandoval & Lee, 2006), or create an expectation for future reciprocity (van der Rijt et al., 2013). Future work might therefore explore the extent to which such factors influence social offloading decisions—as well as the extent to which the findings from our visuospatial memory task generalise to other memory or problem-solving contexts. Second, it is notable that the game Concentration used in the helper videos (i) had substantial overlap with the circle task, in that both tasks required remembering and recalling hidden targets, and (ii) showed predigested information, where participants saw continually updated scores and a clear overall winner. A worthy extension of our work would be to explore the extent to which participants generalise expertise across contexts that have less overlap with the main task, and to what extent the observed effects change according to whether participants receive information via passive observation of behaviours, predigested information, or explicit instructions.

While there are countless examples of cognitive offloading with objects throughout human history (for examples, see Ascher & Ascher, 1981; Best, 1921; Carlson, 1975; Roberts & Roberts, 1996; Schmitt et al., 2014), our unique propensity to outsource cognition onto other humans is indisputably one of the oldest and most widely used instances of this phenomenon. Here, we systematically examined the mechanisms underlying decisions to offload cognitive demand onto others with varying levels of cognitive competency. Our results provide evidence that without expertise being explicitly delineated, people can acquire beliefs about others’ cognitive proficiencies by observing their performance in a different but relevant context and can readily draw upon these beliefs to inform social offloading decisions.

## Supplementary Information

Below is the link to the electronic supplementary material.

Supplementary file1 (PDF 464 KB)

## Data Availability

The datasets generated during the current study are publicly available in the Open Science Framework repository (https://osf.io/teq2x/).

## Code availability

The materials and code for the experiment are publicly available in the Open Science Framework repository (https://osf.io/teq2x/).
