# Supplementary material for: Can you help me? Using others to offload cognition
Source: Mem Cognit. 2024 Aug 22;53(3):946–59. doi: 10.3758/s13421-024-01621-9 (PMC12052855; doi:10.3758/s13421-024-01621-9)
Supplement: Supplementary file 1 — Supplementary file1 (PDF 464 KB) [file 13421_2024_1621_MOESM1_ESM.pdf]

Supplementary Materials for:

Can you help me? Using others to offload cognition

Kristy L. Armitage and Jonathan Redshaw

S1 | Full Instructions

Task Instructions

1. This is a memory game. At the beginning of each trial, you will see lots of white circles that look like this [press space to continue].
2. You will then watch some of the circles turn black and then back to white. These are the target circles [press space to continue].
3. When the target circles have stopped flashing, there will be a five second countdown. Once you see ‘Go!’, your job is to click on as many of the target circles as you can recall, in whatever order you like. Try clicking on some circles. If you click on a circle that is a target, the circle will turn green. If you click on a circle that is not a target, the circle will turn red [press space to continue].
4. You will only be allowed the same number of guesses as the number of targets. For example, if there are 5 targets, you will have 5 guesses. You can see how many guesses you have left in the top right corner of your screen [press space to continue].

Metacognitive prediction measures

1. On some trials, there will be one (1) target circle.
2. On average, how many target circles do you think you will find when there is one (1) target circle? Use the slider to indicate your rating. To submit, click the box beneath the slider (slider ranged from 0 to 1 in increments of 0.1).
3. On some trials, there will be five (5) target circles.
4. On average, how many target circles do you think you will find when there are five (5) target circles? Use the slider to indicate your rating. To submit, click the box beneath the slider (slider ranged from 0 to 5 in increments of 0.1)..
5. On some trials, there will be ten (10) target circles.
6. On average, how many target circles do you think you will find when there are ten (10) target circles? Use the slider to indicate your rating. To submit, click the box beneath the slider (slider ranged from 0 to 10 in increments of 0.1)..

Main task phase 1 (unaided memory)

1. Ready? Press space to start the game.
2. There is one (1) target circle on this trial [press space to start trial]. **Repeated for 9 trials.**

3. Great work! Shortly, you will be redirected to a new screen. Here, you will watch two people play a different game. Please wait…

Memory competence of helpers

1. Searching for players…
2. Two players found! Waiting for photos…
3. Photos received.
4. Click and speak to test audio. P1: “Hey, can you hear me”. P2: “Yes, I can”. P1: Are you ready for me to start the instructions?”. P2: “I’m ready”.
5. A deck of cards will be shuffled and placed face down in rows. These cards look the same on one side but are coloured on the other. Each card has a matching pair.
6. Players will take turns. On each turn, choose two cards to turn over (one at a time). If the cards show the same colour, you will keep the cards.
7. If the cards show different colours, they will be turned face down again.
8. Try to be as fast as possible. The player that has collected the most cards at the end of the game will win. Press ‘start game’ when you are ready to begin.
9. P1: “Do you need to watch the instructions again, or are you ready to start the game?”. P2: “I’m ready to start the game”.
10. Winner: Player 1 (or 2). Press here to end game.
11. Redirecting you back to the first game…

Main task phase 2 (helper-assisted memory)

1. You will soon play the first game again. This time, the people who just finished playing the card game will be watching your screen. You will be able to interact with them and can ask them to help find target circles. On some trials, you will be able to ask Player 1 for help, and on other trials, you will be able to ask Player 2 for help [press space to continue].
2. A picture of the available person will appear in the top left corner of your screen. Here is an example [press space to continue].
3. On each trial, click on as many of the target circles as you can recall. For example, you might remember two target circles [press space to continue].
4. If you get stuck, you can either keep clicking circles, or you can click on the available person [click on the person to continue].
5. If you click on the person, they will be told to finish the remaining guesses for that trial [press space to continue].

6. You can see your total score in the bottom right corner. You and the others will work together. If you find a target circle, your total score will increase by 1. If you ask for help and the other person finds a target circle, your total score will also increase by 1. In this example, you found 2 target circles and the other person found 4 target circles, so your total score increased by 6 [press space to continue].
7. Your total score is important. If your total score at the end of the task places in the top five out of all the participants who are doing the study, you will win a cash prize.
   1st place: $150
   2nd place: $100
   3rd place: $50
   4th place: $50
   5th place: $50.
   If you win a cash prize, you will be contacted via email at the end of data collection to arrange payment. Remember, your total score will increase if either you or the other people find target circles. Please ask the experimenter if you have any questions [press space to continue].
8. Ready? Press space to start the game.
9. Please wait while all players are redirected…
10. There are five (5) target circles on this trial. Player 2 is available. After the countdown, their photo will appear in the corner of the screen. Click on them at any time to ask for help [press space to start trials]. **Repeated for 18 trials.**

S2 | Counterbalancing

Target circles:

A random number generator was used to select the target circles on each trial. The following constraints were used:

(1) A target cannot be located directly next to another target

(2) For one-target trials, the target must be located at least once in each ring across the unaided trials (where there are three 1-target trials in total as in Phase 1) and at least twice in each ring across the aided trials (where there are six 1-target trials in total as in Phase 2)

(3) For five-target and ten-target trials, there must be at least one target in each ring

Main task phase 1 (unaided memory):

The first phase consists of 9 trials (3 easy, 3 medium, 3 hard). To ensure that participants do not see all easy trials first, for example, the order of trial difficulty was arranged in the following sets:

Set 1: Easy, Medium, Hard

Set 2: Medium, Hard, Easy

Set 3: Hard, Easy, Medium

All participants will see Set 1 first, to allow them to experience the task in increasing difficulty. This will then be followed by Set 2 then Set 3 (unaided condition 1) or Set 3 then Set 2 (unaided condition 2). For unaided condition 1, the target circles were determined by following the steps outlined in the target circles sub-section (above). This order was then reversed for unaided condition 2 (such that the target circles in the last hard trial in unaided condition 1 were presented in the reverse order in the first hard trial in unaided condition 2, with the same process repeated for all trials), to account for any possible order effects.

Memory competence of helpers:

Four versions of the Concentration game were created. In each version, the players were either both male or both female, and either Player 1 or Player 2 won the game. All other variables were kept constant across versions.

Version 1: Female players, Player 1 wins

Version 2: Female players, Player 2 wins (same pictures and voice recordings as Version 1)

Version 3: Male players, Player 1 wins (exact copy of Version 1 but with male pictures/voices)

Version 4: Male players, Player 2 wins (exact copy of Version 2 but with male pictures/voices, same pictures and voice recordings as Version 3)

Main task phase 2 (helper-assisted memory):

In the third phase, there were six possible trial types:

(1) Strong-memory helper available, one target

(2) Strong-memory helper available, five targets

(3) Strong-memory helper available, ten targets

(4) Weak-memory helper available, one target

(5) Weak-memory helper available, five targets

(6) Weak-memory helper available, ten targets

Across 18 trials, participants will see each of these possible trial types three times. The order of trials was decided using the following constraints:

1. Trial were organised into blocks of three, each made up of the six types of trials in different orders

2. Trials of the same type are separated by at least two other types of trials.

3. The same helper can only appear on a maximum of two consecutive trials.

For aided condition 1, the target circles were determined by following the steps outlined in the target circles sub-section (above). To create aided condition 2, the order of trials was reversed, as well as the type of trial (strong-memory helper 1 target became weak-memory helper 1 target and vice versa, repeated for all trial types). We also reversed the order of target circles, to account for any possible order effects.

Overall:

We combined the above counterbalancing measures to create 8 conditions:

1. Unaided Order 1, Aided Order 1 (Female) – Player 1
2. Unaided Order 1, Aided Order 1 (Male) – Player 2
3. Unaided Order 1, Aided Order 2 (Female) – Player 2
4. Unaided Order 1, Aided Order 2 (Male) – Player 1
5. Unaided Order 2, Aided Order 1 (Female) – Player 2
6. Unaided Order 2, Aided Order 1 (Male) – Player 1
7. Unaided Order 2, Aided Order 2 (Female) – Player 1
8. Unaided Order 2, Aided Order 2 (Male) – Player 2

S3 | Phase 1 Accuracy Analyses

Phase 1

P1 Accuracy (Proportion)

Effects	*df*	*F*	*p*	η_p^2
Base model (-2LL = -343.42)				
Age (control)	1, 957	1.80	.180	- < .01
**Difficulty**	1, 957	**48.93**	**< .001**	**- .05**
Predicted (mean-centred)	1, 957	1.54	.215	- < .01
**Trial**	**1, 957**	**4.51**	**.034**	**< .01**
Interaction model 1 (-2LL = -344.32)				
Difficulty x predicted (mean-centred)	1, 596	0.89	.345	< .01

Note. Significant focal effects are bolded. A Likelihood Ratio Test was used to compare the goodness of fit for interaction model 1 (-2 log likelihood values can be found in the table above). The base model was not outperformed by interaction model 1, $\chi^2(1) = 0.90$, p = .343.

S4 | Phase 2 Offloading (Binary) Analyses

Offloading (Binary)

Effects	*df*	χ^2	*p*	*w*
Base model (-2LL = 1356.31)				
**Age (control)**	**1, 1315**	**5.23**	**.022**	**0.21**
**Difficulty**	**1, 1315**	**205.15**	**< .001**	**1.31**
**Helper memory**	**1, 1315**	**104.61**	**< .001**	**0.93**
Trial	1, 1315	0.07	.786	- 0.02
**P1 accuracy (proportion)**	**1, 1315**	**11.84**	**.001**	**- 0.31**
Predicted accuracy	1, 1315	0.02	.902	0.01
Interaction model 1 (-2LL = 1355.22)				
Difficulty x helper memory	1, 1314	1.06	.303	**0.09**
Interaction model 2 (-2LL = 1352.28)				
**Difficulty x p1 accuracy**	**1, 1314**	**4.00**	**.046**	**0.18**
Interaction model 3 (-2LL = 1356.30)				
Helper memory x p1 accuracy	1, 1314	0.00	.948	< 0.01
Interaction model 4 (-2LL = 1356.27)				
Predicted accuracy x difficulty	1, 1314	0.04	.844	0.02
Interaction model 5 (-2LL = 1355.97)				
Predicted accuracy x helper memory	1, 1314	0.33	.566	0.05
Interaction model 6 (-2LL = 1355.18)				
Predicted accuracy x p1 accuracy	1, 1314	1.13	.287	0.10

Note. Significant focal effects are bolded. Helper memory was coded as weak-memory = 0, strong-memory = 1. Likelihood Ratio Tests were used to compare the goodness of fit for all interaction models (see -2 log likelihood values in the table above). The base model was outperformed by interaction model 2, $\chi^2(1) = 4.03$, $p = .045$, but was not outperformed by interaction model 1, $\chi^2(1) = 1.09$, $p = .296$, interaction model 3, $\chi^2(1) = 0.01$, $p = .920$, interaction model 4, $\chi^2(1) = 0.04$, $p = 8.41$, interaction model 5, $\chi^2(1) = 0.34$, $p = .560$, or interaction model 6, $\chi^2(1) = 1.13$, $p = .288$. Follow-up analyses for significant interaction terms can be found below.

Interaction Model 2 (Difficulty x P1 Accuracy) Follow-up

	df	χ^2	*p*	*w*
**Effect of p1 accuracy on 5-target trials**	**1, 1314**	**6.18**	**.026**	**0.31**
**Effect of p1 accuracy on 10-target trials**	**1, 1314**	**11.88**	**.001**	**0.23**

Note. Significant focal effects are bolded. Bonferroni corrections are applied.

Offloading (Binary) – Effect of P2 Unaided Accuracy (Proportional Accuracy)

Effects	*df*	χ^2	*p*	*w*
Base model (-2 LL = 1351.63)				
**Age (control)**	**1, 1316**	**8.12**	**.004**	**0.26**
**Difficulty**	**1, 1316**	**257.03**	**< .001**	**1.46**
**Helper memory**	**1, 1316**	**98.59**	**< .001**	**0.91**
Trial	1, 1316	0.16	.685	- 0.04
**P2 unaided accuracy**	**1, 1316**	**15.80**	**< .001**	**- 0.36**
Interaction model 1 (-2 LL = 1339.47)				
**P2 unaided accuracy x difficulty**	**1, 1315**	**11.81**	**.001**	**0.31**
Interaction model 2 (-2 LL = 1349.54)				
P2 unaided accuracy x helper memory	1, 1315	2.07	.151	0.13

Note. Significant focal effects are bolded. Helper memory was coded as weak-memory = 0, strong-memory = 1. Likelihood Ratio Tests were used to compare the goodness of fit for all interaction models (see -2 log likelihood values in the table above). The base model was outperformed by interaction model 1, $\chi^2(1) = 12.16$, $p < .001$, and interaction model 2, $\chi^2(1) = 10.07$, $p = .002$. Follow-up analyses for significant interaction terms can be found below.

Interaction Model 1 (P2 Unaided Accuracy x Difficulty) Follow-up

	df	χ^2	*p*	*w*
Effect of unaided accuracy – 5-target	1, 1315	2.74	.197	0.15
**Effect of unaided accuracy - 10-target**	**1, 1315**	**25.11**	**< .001**	**0.46**

Note. Significant focal effects are bolded. Bonferroni corrections are applied.

Offloading (Binary) – Effect of P2 Unaided Accuracy (Total Accuracy)

Effects	*df*	χ^2	*p*	*w*
Base model (-2LL = 1210.46)				
Age (control)	1, 1316	2.79	.095	**0.15**
**Difficulty**	**1, 1316**	**280.86**	**< .001**	**1.53**
**Helper memory**	**1, 1316**	**82.75**	**< .001**	**0.83**
Trial	1, 1316	0.07	.795	- 0.02
**P2 unaided accuracy (total)**	**1, 1316**	**128.99**	**< .001**	**- 1.04**
Interaction model 1 (-2LL = 1180.58)				
**P2 unaided accuracy x difficulty**	**1, 1315**	**27.75**	**< .001**	**0.48**
Interaction model 2 (-2LL = 1208.87)				
P2 unaided accuracy x helper memory	1, 1315	1.59	.208	0.12

Note. Significant focal effects are bolded. Helper memory was coded as weak-memory = 0, strong-memory = 1. Likelihood Ratio Tests were used to compare the goodness of fit for all interaction models (see -2 log likelihood values in the table above). The base model was outperformed by interaction model 1, $\chi^2(1) = 29.88$, $p < .001$, but not interaction model 2, $\chi^2(1) = 1.59$, $p = .207$. Follow-up analyses for significant interaction terms can be found below.

Interaction Model 1 (P2 Unaided Accuracy x Difficulty) Follow-up

	df	χ^2	*p*	*w*
**Effect of unaided accuracy – 5-target**	**1, 1315**	**113.02**	**< .001**	**- 0.97**
**Effect of unaided accuracy - 10-target**	**1, 1315**	**34.77**	**< .001**	**- 0.54**

Note. Significant focal effects are bolded. Bonferroni corrections are applied.

S5 | Phase 2 Offloading Threshold Analyses

Offloading (Threshold) – Effect of P1 Accuracy

Effects	*df*	*F*	*p*	η_p^2
Base model (-2LL = -761.82)				
Age (control)	1, 1315	2.12	.146	- < .01
**Difficulty**	**1, 1315**	**275.45**	**< .001**	**- .17**
**Helper memory**	**1, 1315**	**229.28**	**< .001**	**- .15**
**Trial**	**1, 1315**	**6.51**	**.011**	**- < .01**
**P1 Accuracy (proportion)**	**1, 1315**	**15.58**	**< .001**	**.01**
Predicted Accuracy	1, 1315	0.66	.417	- < .01
Interaction model 1 (-2LL = -765.27)				
Difficulty x helper memory	1, 1314	3.46	.063	< .01
Interaction model 2 (-2LL = -776.79)				
**Difficulty x p1 accuracy**	**1, 1314**	**15.06**	**< .001**	**.01**
Interaction model 3 (-2LL = -763.55)				
Helper memory x p1 accuracy	1, 1314	1.73	.189	< .01
Interaction model 4 (-2LL = -763.52)				
Predicted accuracy x difficulty	1, 1314	1.71	.192	< .01
Interaction model 5 (-2LL = -761.95)				
Predicted accuracy x helper memory	1, 1314	0.13	.717	< .01
Interaction model 6 (-2LL = -764.69)				
Predicted accuracy x p1 accuracy	1, 1314	2.87	.090	< .01

Note. Significant focal effects are bolded. Helper memory was coded as weak-memory = 0, strong-memory = 1. Likelihood Ratio Tests were used to compare the goodness of fit for all interaction models (see -2 log likelihood values in the table above). The base model was outperformed by interaction model 2, $\chi^2(1) = 14.97$, $p < .001$, but not interaction model 1, $\chi^2(1) = 3.45$, $p = .063$, interaction model 3, $\chi^2(1) = 1.73$, $p = .188$, interaction model 4, $\chi^2(1) = 1.70$, $p = .192$, interaction model 5, $\chi^2(1) = 0.13$, $p = .718$, and interaction model 6, $\chi^2(1) = 2.87$, $p = .192$. Follow-up analyses for significant interaction terms can be found below.

Interaction Model 2 (Difficulty x P1 Accuracy) Follow-up

	df	*F*	*p*	η_p^2
Effect of p1 acc on 5-target trials	1, 1314	5.04	.050	< .01
**Effect of p1 acc on 10-target trials**	**1, 1314**	**30.39**	**< .001**	**.02**

Note. Significant focal effects are bolded. Bonferroni corrections are applied.

Offloading (Threshold) – Effect of P2 Accuracy (Proportional Accuracy)

Effects	*df*	*F*	*p*	η_p^2
Base model (-2LL = -749.93)				
Age (control)	1, 1316	3.42	.065	**- < .01**
**Difficulty**	**1, 1316**	**423.59**	**< .001**	**- .24**
**Helper memory**	**1, 1316**	**219.79**	**< .001**	**- .14**
**Trial**	**1, 1316**	**6.17**	**.013**	**- < .01**
**P2 unaided accuracy (proportion)**	**1, 1316**	**4.60**	**.032**	**+ < .01**
Interaction model 1 (-2LL = -757.25)				
**P2 unaided accuracy x difficulty**	**1, 1315**	**7.34**	**.007**	**.01**
Interaction model 2 (-2LL = -758.16)				
**P2 unaided accuracy x helper memory**	**1, 1315**	**8.27**	**.004**	**.01**

Note. Significant focal effects are bolded. Helper memory was coded as weak-memory = 0, strong-memory = 1. Likelihood Ratio Tests were used to compare the goodness of fit for all interaction models (see -2 log likelihood values in the table above). The base model was outperformed by interaction model 1, $\chi^2(1) = 7.32$, $p = .026$, and interaction model 2, $\chi^2(1) = 8.23$, $p = .016$. Follow-up analyses for significant interaction terms can be found below.

Interaction Model 1 (P2 Unaided Accuracy x Difficulty) Follow-up

	df	*F*	*p*	η_p^2
Effect of unaided score – 5-target	1, 1315	0.02	> .999	+ < .01
**Effect of unaided score - 10-target**	**1, 1315**	**11.77**	**.001**	**- .01**

Note. Significant focal effects are bolded. Bonferroni corrections are applied.

Interaction Model 2 (P2 Unaided Accuracy x Helper Memory) Follow-up

	df	*F*	*p*	η_p^2
**Effect of unaided score – weak-memory**	**1, 1315**	**12.72**	**.001**	**.01**
Effect of unaided score – strong-memory	1, 1315	0.00	> .999	< .01

Note. Significant focal effects are bolded. Bonferroni corrections are applied.

Offloading (Threshold) – Effect of P2 Accuracy (Total Accuracy)

Effects	*df*	*F*	*p*	η_p^2
Base model (-2LL = -1264.85)				
Age (control)	1, 1316	0.01	.903	- < .01
**Difficulty**	**1, 1316**	**1016.51**	**< .001**	**- .44**
**Helper memory**	**1, 1316**	**171.09**	**< .001**	**- .12**
**Trial**	**1, 1316**	**9.10**	**.003**	**- .01**
**P2 unaided accuracy (total)**	**1, 1316**	**643.39**	**< .001**	**.33**
Interaction model 1 (-2LL = -1291.44)				
**P2 unaided accuracy x difficulty**	**1, 1315**	**26.83**	**< .001**	**.02**
Interaction model 2 (-2LL = -1288.52)				
**P2 unaided accuracy x helper memory**	**1, 1315**	**23.68**	**< .001**	**.02**

Note. Significant focal effects are bolded. Helper memory was coded as weak-memory = 0, strong-memory = 1. Likelihood Ratio Tests were used to compare the goodness of fit for all interaction models (see -2 log likelihood values in the table above). The base model was outperformed by interaction model 1, $\chi^2(1) = 26.64$, $p < .001$ and interaction model 2, $\chi^2(1) = 23.72$, $p < .001$. Follow-up analyses for significant interaction terms can be found below.

Interaction Model 1 (P2 Unaided Accuracy x Difficulty) Follow-up

	df	*F*	*p*	η_p^2
**Effect of unaided score – 5-target**	**1, 1315**	**380.11**	**< .001**	**.22**
**Effect of unaided score – 10-target**	**1, 1315**	**396.24**	**< .001**	**.23**

Note. Significant focal effects are bolded. Bonferroni corrections are applied.

Interaction Model 2 (P2 Unaided Accuracy x Helper memory) Follow-up

	df	*F*	*p*	η_p^2
**Effect of unaided score – weak-memory**	**1, 1315**	**258.53**	**< .001**	**.16**
**Effect of unaided score – strong-memory**	**1, 1315**	**535.22**	**< .001**	**.29**

Note. Significant focal effects are bolded. Bonferroni corrections are applied.

S6 | Phase 2 Accuracy Analyses

Phase 2 Combined Accuracy (Binary Offloading IV)

Effects	*df*	*F*	*p*	η_p^2
Base model (-2LL = -854.86)				
**Age (control)**	**1, 1315**	**7.55**	**.006**	**- < .01**
**Difficulty**	**1, 1315**	**56.73**	**< .001**	**- .04**
**Helper memory**	**1, 1315**	**315.03**	**< .001**	**.19**
**Trial**	**1, 1315**	**11.13**	**.001**	**< .01**
Offloading (binary)	1, 1315	3.60	.058	- < .01
**P1 accuracy**	**1, 1315**	**25.35**	**< .001**	**.02**
Interaction model 1 (-2LL = -869.65)				
**Difficulty x offloading (binary)**	**1, 1314**	**14.87**	**< .001**	**.01**
Interaction model 2 (-2LL = -948.67)				
**Helper memory x offloading (binary)**	**1, 1314**	**97.14**	**< .001**	**.07**
Interaction model 3 (-2LL = -978.18)				
**Difficulty x offloading (binary) x helper memory**	**3, 1311**	**37.67**	**< .001**	**.08**
Interaction model 4 (-2LL = -858.75)				
**P1 accuracy x offloading (binary)**	**1, 1314**	**3.90**	**.049**	**< .01**
Interaction model 5 (-2LL = -955.25)				
**P1 accuracy x helper memory x offloading (binary)**	**2, 1312**	**50.00**	**< .001**	**.07**

Note. Significant focal effects are bolded. Helper memory was coded as weak-memory = 0, strong-memory = 1. Offloading (binary) was coded as no offloading = 0, offloading = 1. Likelihood Ratio Tests were used to compare the goodness of fit for all interaction models (see -2 log likelihood values in the table above). The base model was outperformed by interaction model 1, $\chi^2(1) = 14.79$, $p < .001$, interaction model 2, $\chi^2(1) = 93.81$, $p < .001$, interaction model 3, $\chi^2(4) = 123.32$, *p* <. 001, interaction model 4, $\chi^2(1) = 3.89$, $p = .049$, and interaction model 5, $\chi^2(3) = 100.39$, $p < .001$. Follow-up analyses for significant interaction terms can be found below.

Interaction Model 1 (Difficulty x Offloading (Binary)) Follow-up

	df	*F*	*p*	η_p^2
**Effect of offloading on 5-target trials**	**1, 1314**	**13.15**	**.001**	**- .01**
Effect of offloading on 10-target trials	1, 1314	3.92	.096	< .01

Note. Significant focal effects are bolded. Bonferroni corrections are applied. Offloading (binary) was coded as no offloading = 0, offloading = 1.

Interaction Model 2 (Helper Memory x Offloading (Binary)) Follow-up

	df	*F*	*p*	η_p^2
**Offloading (binary) – weak-memory**	**1, 1314**	**53.34**	**< .001**	**- .04**
**Offloading (binary) – strong-memory**	**1, 1314**	**30.98**	**< .001**	**.02**

Note. Significant focal effects are bolded. Bonferroni corrections are applied. Offloading (binary) was coded as no offloading = 0, offloading = 1.

Interaction Model 3 (Difficulty x Offloading (Binary) x Helper Memory) Follow-up

	df	*F*	*p*	η_p^2
**Effect of offloading on 5-target trials – weak-memory**	**1, 1311**	**43.34**	**< .001**	**- .03**
**Effect of offloading on 5-target trials – strong-memory**	**1, 1311**	**8.64**	**.013**	**.01**
Effect of offloading on 10-target trials – weak-memory	1, 1311	2.07	.602	- < .01
**Effect of offloading on 10-target trials – strong-memory**	**1, 1311**	**22.73**	**< .001**	**.02**

Note. Significant focal effects are bolded. Bonferroni corrections are applied. Offloading (binary) was coded as no offloading = 0, offloading = 1.

Interaction Model 4 (P1 Accuracy x Offloading (Binary)) Follow-up

	df	*F*	*p*	η_p^2
**P1 accuracy – no offloading**	**1, 1314**	**6.36**	**.024**	**< .01**
**P1 accuracy – offloading**	**1, 1314**	**27.44**	**< .001**	**.02**

Note. Significant focal effects are bolded. Bonferroni corrections are applied.

Interaction Model 5 (P1 Accuracy x Helper Memory x Offloading (Binary)) Follow-up

	df	*F*	*p*	η_p^2
**P1 accuracy, weak-memory, no offloading**	**1, 1312**	**49.85**	**< .001**	**.04**
P1 accuracy, weak-memory, offloading	1, 1312	2.63	.420	< .01
P1 accuracy, strong-memory, no offloading	1, 1312	3.21	.294	< .01
P1 accuracy, strong-memory, offloading	1, 1312	4.50	.136	< .01

Note. Significant focal effects are bolded. Bonferroni corrections are applied.

Phase 2 Combined Accuracy (Offloading Threshold IV)

Effects	*df*	*F*	*p*	η_p^2
Base model (-2LL = -851.34)				
**Age (control)**	**1, 1315**	**8.07**	**.005**	**- .01**
**Difficulty**	**1, 1315**	**73.51**	**< .001**	**- .05**
**Helper memory**	**1, 1315**	**274.89**	**< .001**	**.17**
**Trial**	**1, 1315**	**11.04**	**.001**	**.01**
Offloading threshold	1, 1315	0.08	.784	< .01
**P1 accuracy**	**1, 1315**	**26.84**	**< .001**	**.02**
Interaction model 1 (-2LL = 880.38)				
**Difficulty x offloading threshold**	**1, 1314**	**29.38**	**< .001**	**.02**
Interaction model 2 (-2LL = -1013.72)				
**Helper memory x offloading threshold**	**1, 1314**	**172.60**	**< .001**	**.12**
Interaction model 3 (-2LL = -1045.99)				
**Difficulty x offloading threshold x helper memory**	**2, 1312**	**88.11**	**< .001**	**.21**
Interaction model 4 (-2LL = -857.17)				
**P1 accuracy x offloading threshold**	**1, 1314**	**5.84**	**.016**	**< .01**
Interaction model 5 (-2LL = -964.41)				
**P1 accuracy x helper memory x offloading threshold**	**1, 1313**	**111.46**	**< .001**	**.08**

Note. Significant focal effects are bolded. Helper memory was coded as weak-memory = 0, strong-memory = 1. Offloading (binary) was coded as no offloading = 0, offloading = 1. Likelihood Ratio Tests were used to compare the goodness of fit for all interaction models (see -2 log likelihood values in the table above). The base model was outperformed by interaction model 1, $\chi^2(1) = 29.04$, $p < .001$, interaction model 2, $\chi^2(1) = 162.38$, $p < .001$, interaction model 3, $\chi^2(3) = 194.65$, $p <. 001$, interaction model 4, $\chi^2(1) = 5.83$, $p = .016$, and interaction model 5, $\chi^2(3) = 113.07$, $p < .001$. Follow-up analyses for significant interaction terms can be found below.

Interaction Model 1 (Difficulty x Offloading Threshold) Follow-up

	df	*F*	*p*	η_p^2
**Effect of offloading on 5-target trials**	**1, 1314**	**10.92**	**.002**	**.01**
**Effect of offloading on 10-target trials**	**1, 1314**	**13.81**	**< .001**	**- .01**

Note. Significant focal effects are bolded. Bonferroni corrections are applied.

Interaction Model 2 (Helper Memory x Offloading Threshold) Follow-up

	df	*F*	*p*	η_p^2
**Offloading (threshold) – weak-memory**	**1, 1314**	**86.83**	**< .001**	**.06**
**Offloading (threshold) – strong-memory**	**1, 1314**	**52.41**	**< .001**	**- .04**

Note. Significant focal effects are bolded. Bonferroni corrections are applied.

Interaction Model 3 (Difficulty x Offloading Threshold x Helper Memory) Follow-up

	df	*F*	*p*	η_p^2
**Effect of offloading on 5-target trials – weak-memory**	**1, 1312**	**114.68**	**< .001**	**.08**
**Effect of offloading on 5-target trials – strong-memory**	**1, 1312**	**26.65**	**< .001**	**- .02**
**Effect of offloading on 10-target trials – weak-memory**	**1, 1312**	**6.54**	**.043**	**< .01**
**Effect of offloading on 10-target trials – strong-memory**	**1, 1312**	**61.52**	**< .001**	**- .04**

Note. Significant focal effects are bolded. Bonferroni corrections are applied.

Interaction Model 4 (P1 Accuracy x Offloading Threshold) Follow-up

	df	*SE*	*t*	*p*
Effect of p1 accuracy at 1SD below offloading threshold mean	1314	.073	1.54	.491
**Effect of p1 accuracy at offloading threshold mean**	**1314**	**.050**	**4.16**	**< .001**
**Effect of p1 accuracy at 1SD above offloading threshold mean**	**1314**	**.054**	**5.70**	**< .001**

Note. Significant focal effects are bolded. Bonferroni corrections are applied.

Interaction Model 5 (P1 Accuracy x Helper Memory x Offloading Threshold) Follow-up

	df	*F*	*p*	η_p^2
Effect of p1 accuracy at 1SD below offloading threshold mean – strong-memory	1, 595	2.28	.790	< .01
**Effect of p1 accuracy at offloading threshold mean – strong-memory**	**1, 595**	**11.49**	**.004**	**.02**
**Effect of p1 accuracy at 1SD above offloading threshold mean – strong-memory**	**1, 595**	**18.68**	**< .001**	**.03**
Effect of p1 accuracy at 1SD below offloading threshold mean – weak-memory	1, 595	0.70	> .999	< .01
**Effect of p1 accuracy at offloading threshold mean – weak-memory**	**1, 595**	**8.75**	**.019**	**.01**
**Effect of p1 accuracy at 1SD above offloading threshold mean – weak-memory**	**1, 595**	**18.20**	**< .001**	**.03**

Note. Significant focal effects are bolded. Bonferroni corrections are applied.

S7 | Cross-Phase Accuracy Analyses

Cross-Phase Accuracy Comparison (Proportional Accuracy)

Effects	*df*	*F*	*p*	η_p^2
Base model (-2LL = -566.98)				
**Age**	**1, 2037**	**10.97**	**.001**	**- .01**
**Difficulty**	**1, 2037**	**122.80**	**< .001**	**- .06**
**Phase**	**1, 2037**	**410.32**	**< .001**	**+ .17**
**Trial**	**1, 2037**	**5.72**	**.017**	**+ < .01**
Interaction model 1 (-2LL = -567.10)				
Difficulty x phase	1, 2036	0.12	.728	< .01

Note. Significant focal effects are bolded. Phase was coded as 1 = phase 1, 2 = phase 2. Likelihood Ratio Tests were used to compare the goodness of fit for all interaction models (see -2 log likelihood values in the table above). The base model was not outperformed by interaction model 1, $\chi^2(1) = 0.12$, $p = .729$.

Cross-Phase Accuracy Comparison (Total Accuracy)

Effects	*df*	*F*	*p*	η_p^2
Base model (-2LL = 7372.97) - 7				
**Age**	**1, 2037**	**6.40**	**.012**	**- < .01**
**Difficulty**	**1, 2037**	**570.08**	**< .001**	**.22**
Phase	1, 2037	0.56	.456	< .01
Trial	1, 2037	1.56	.211	< .01
Interaction model 1 (-2LL = 7325.71) - 8				
**Difficulty x phase**	**1, 2036**	**47.81**	**< .001**	**.02**

Note. Significant focal effects are bolded. Phase was coded as 1 = phase 1, 2 = phase 2. Likelihood Ratio Tests were used to compare the goodness of fit for all interaction models (see -2 log likelihood values in the table above). The base model was outperformed by interaction model 1, $\chi^2(1) = 47.26$, $p < .001$.

Interaction Model 1 (Difficulty x Phase) Follow-up

	df	*F*	*p*	η_p^2
**Effect of phase – 5-target**	**1, 2036**	**29.41**	**< .001**	**.01**
**Effect of phase – 10-target**	**1, 2036**	**18.97**	**< .001**	**- .01**

Note. Significant focal effects are bolded. Bonferroni corrections are applied.

S8 | Participant Sex Effects

We explored whether any significant offloading effects varied by participant sex.

Offloading (Binary)

Effects	*df*	χ^2	*p*	*w*
Base model				
Age (control)	1, 1315	5.20	.023	0.21
Difficulty	1, 1315	205.22	< .001	1.31
Helper memory	1, 1315	104.61	< .001	0.93
Trial	1, 1315	0.07	.786	0.02
P1 accuracy (proportion)	1, 1315	11.80	.001	0.31
Predicted accuracy	1, 1315	0.01	.930	0.01
Sex	1, 1315	0.14	.712	0.03
Interaction model 1				
Difficulty x helper memory	1, 1311	1.11	.291	0.10
Difficulty x helper memory x sex	3, 1311	0.56	.640	0.07
Interaction model 2				
Difficulty x p1 accuracy	1, 1312	6.51	.011	0.23
**Difficulty x p1 accuracy x sex**	**2, 1312**	**6.63**	**.036**	**0.24**

Note. Significant sex effects are bolded. Helper memory was coded as weak-memory = 0, strong-memory = 1. Sex was coded as male = 0, female = 1.

Interaction Model 2 (Difficulty x P1 Accuracy x Sex) Follow-up

	df	χ^2	*p*	*w*
Accuracy slope for male, 5-targets	1, 1312	0.00	> .999	- < 0.01
Accuracy slope for male, 10-targets	1, 1312	6.21	.051	- 0.32
**Accuracy slope for female, 5-targets**	1, 1312	**12.00**	**.001**	**- 0.45**
**Accuracy slope for female, 10-targets**	1, 1312	**19.50**	**< .001**	**- 0.57**

Note. Significant sex effects are bolded. Bonferroni corrections are applied. Sex was coded as male = 0, female = 1.

Offloading (Threshold)

Effects	*df*	*F*	*p*	η_p^2
Base model				
Age (control)	1, 1314	2.57	.109	< .01
Difficulty	1, 1314	282.32	< .001	.18
Helper memory	1, 1314	219.80	< .001	.14
Trial	1, 1314	6.08	.014	< .01
P1 accuracy (proportion)	1, 1314	16.90	< .001	.01
Predicted accuracy	1, 1314	0.53	.466	< .01
P2 accuracy	1, 1314	5.70	.017	< .01
Sex	1, 1314	0.76	.384	< .01
Interaction model 1				
Difficulty x p1 accuracy	1, 1428	26.02	< .001	.02
**Difficulty x p1 accuracy x sex**	**2, 1428**	**5.68**	**.004**	**.01**
Interaction model 2				
P2 accuracy x difficulty	1, 1311	6.71	.010	.01
P2 accuracy x difficulty x sex	2, 1311	0.28	.758	< .01
Interaction model 4				
P2 accuracy x helper memory	1, 1311	8.04	.005	.01
P2 accuracy x helper memory x sex	2, 1311	1.28	.278	< .01

Note. Significant sex effects are bolded. Helper memory was coded as weak-memory = 0, strong-memory = 1. Sex was coded as male = 0, female = 1.

Interaction Model 1 (Difficulty x P1 Accuracy x Sex) Follow-up

	df	*F*	*p*	η_p^2
Accuracy slope for male, 5-targets	1, 1428	0.07	> .999	< .01
**Accuracy slope for male, 10-targets**	**1, 1428**	**24.34**	**< .001**	**.02**
**Accuracy slope for female, 5-targets**	**1, 1428**	**22.49**	**< .001**	**.02**
**Accuracy slope for female, 10-targets**	**1, 1428**	**73.12**	**< .001**	**.05**

Note. Significant sex effects are bolded. Bonferroni corrections are applied. Sex was coded as male = 0, female = 1.

Effect of participant sex and helper sex on offloading (binary)

Effects	*df*	χ^2	*p*	*w*
Age (control)	1, 1317	6.63	.010	0.24
Difficulty	1, 1317	260.04	< .001	1.47
Helper memory	1, 1317	104.30	< .001	0.93
Trial	1, 1317	0.07	.796	0.02
Sex	1, 1317	0.21	.647	0.04
Helper sex	1, 1317	4.20	.040	0.19
**Sex x helper sex**	**1, 1317**	**4.64**	**.031**	**0.20**

Note. Significant sex effects are bolded. Sex was coded as male = 0, female = 1. Helper sex was coded as male = 0, female = 1.

Interaction (Sex x Helper Sex) Follow-up

	df	χ^2	*p*	*w*
**Effect of helper sex for male participants**	**1, 1317**	**8.74**	**.006**	**0.38**
Effect of helper sex for female participants	1, 1317	0.01	> .999	- 0.01

Note. Significant sex effects are bolded. Bonferroni corrections are applied. Sex was coded as male = 0, female = 1. Helper sex was coded as male = 0, female = 1.

S9 | Helper Sex Effects

We explored whether any significant offloading effects varied by helper sex.

Offloading (Binary)

Effects	*df*	χ^2	*p*	*w*
Base model				
Age (control)	1, 1315	5.30	.021	0.21
Difficulty	1, 1315	206.69	< .001	1.31
Helper memory	1, 1315	104.63	< .001	0.93
Trial	1, 1315	0.07	.786	0.02
P1 accuracy (proportion)	1, 1315	11.00	.001	0.30
Predicted accuracy	1, 1315	0.00	.982	< 0.01
Helper sex	1, 1315	3.28	.070	0.17
Interaction model 1				
Difficulty x helper memory	1, 1311	1.72	.190	0.12
Difficulty x helper memory x helper sex	2, 1311	6.92	.074	0.24

Note. Significant helper sex effects are bolded. Helper memory was coded as weak-memory = 0, strong-memory = 1. Helper sex was coded as male = 0, female = 1.

Offloading (Threshold)

Effects	*df*	*F*	*p*	η_p^2
Base model				
Age (control)	1, 1314	2.59	.108	< .01
Difficulty	1, 1314	282.71	< .001	.18
Helper memory	1, 1314	219.76	< .001	.14
Trial	1, 1314	6.08	.014	< .01
P1 accuracy (proportion)	1, 1314	16.51	< .001	.01
Predicted accuracy	1, 1314	0.53	.469	< .01
P2 unaided accuracy (proportion)	1, 1314	5.78	.016	< .01
Helper sex	1, 1314	0.54	.461	- < .01
Interaction model 1				
Difficulty x p1 accuracy	1, 1428	25.79	< .001	.02
**Difficulty x p1 accuracy x helper sex**	**2, 1428**	**4.75**	**.009**	**.01**
Interaction model 2				
Difficulty x p2 accuracy	1, 1311	5.15	.024	< .01
**Difficulty x p2 accuracy x helper sex**	**2, 1311**	**7.02**	**.001**	**.01**
Interaction model 3				
P2 accuracy x helper memory	1, 1311	7.35	.007	.01
**P2 accuracy x helper memory x helper sex**	**2, 1311**	**7.68**	**.001**	**.01**

Note. Significant helper sex effects are bolded. Helper memory was coded as weak-memory = 0, strong-memory = 1. Helper sex was coded as male = 0, female = 1.

Interaction model 1 (Difficulty x P1 Accuracy x Helper Sex) Follow-up

Effects	*df*	*F*	*p*	η_p^2
**P1 accuracy slope, male helper, 5-targets**	**1, 1311**	**11.74**	**.002**	**.01**
**P1 accuracy slope, male helper, 10-targets**	**1, 1311**	**34.43**	**< .001**	**.03**
P1 accuracy slope, female helper, 5-targets	**1, 1311**	0.11	> .999	< .01
**P1 accuracy slope, female helper, 10-targets**	**1, 1311**	**13.92**	**< .001**	**.01**

Note. Significant helper sex effects are bolded. Bonferroni corrections are applied. Helper sex was coded as male = 0, female = 1.

Interaction model 2 (Difficulty x P2 Accuracy x Helper Sex) Follow-up

Effects	*df*	*F*	*p*	η_p^2
P2 accuracy slope, male helper, 5-targets	1, 1311	5.75	.067	- < .01
**P2 accuracy slope, male helper, 10-targets**	**1, 1311**	**23.54**	**< .001**	**- .02**
P2 accuracy slope, female helper, 5-targets	1, 1311	3.85	.150	< .01
P2 accuracy slope, female helper, 10-targets	1, 1311	0.27	> .999	- < .01

Note. Significant helper sex effects are bolded. Bonferroni corrections are applied. Helper sex was coded as male = 0, female = 1.

Interaction model 3 (P2 Accuracy x Helper Memory x Helper Sex) Follow-up

Effects	*df*	*F*	*p*	η_p^2
**P2 accuracy slope, male helper, weak-memory**	**1, 1311**	**24.82**	**< .001**	**.02**
**P2 accuracy slope, male helper, strong-memory**	**1, 1311**	**6.72**	**.038**	**.01**
P2 accuracy slope, female helper, weak-memory	**1, 1311**	0.93	.> .999	< .01
P2 accuracy slope, female helper, strong-memory	1, 1311	4.72	.120	< .01

Note. Significant helper sex effects are bolded. Bonferroni corrections are applied. Helper sex was coded as male = 0, female = 1.

S10 | Player Winner Effects

We explored whether any significant offloading effects varied by which player won the game.

Offloading (Binary)

Effects	*df*	χ^2	*p*	*w*
Base model				
Age (control)	1, 1315	5.38	.020	0.21
Difficulty	1, 1315	205.98	< .001	1.31
Helper memory	1, 1315	104.63	< .001	0.93
Trial	1, 1315	0.07	.786	0.02
P1 accuracy (proportion)	1, 1315	11.28	.001	0.31
Predicted accuracy	1, 1315	0.02	.887	0.01
Winner	1, 1315	1.48	.224	0.11
Interaction model 1				
Difficulty x helper memory	1, 1311	2.19	.139	0.14
**Difficulty x helper memory x winner**	**3, 1311**	**2.92**	**.033**	**0.16**

Note. Significant winner effects are bolded. Helper memory was coded as weak-memory = 0, strong-memory = 1.

Interaction model 1 (Difficulty x Helper Memory x Winner) Follow-up

Effects	*df*	χ^2	*p*	*w*
5-targets, weak memory – effect of winner	1, 1311	0.09	> .999	0.03
5-targets, strong memory – effect of winner	1, 1311	3.41	.260	0.17
10-targets, weak memory – effect of winner	1, 1311	0.35	> .999	0.05
10-targets, strong memory – effect of winner	1, 1311	5.56	.074	0.22

Note. Significant winner effects are bolded. Bonferroni corrections are applied.

Offloading (Threshold)

Effects	*df*	*F*	*p*	η_p^2
Base model				
Age (control)	1, 1314	2.70	.101	< .01
Difficulty	1, 1314	282.55	< .001	.18
Helper memory	1, 1314	219.72	< .001	.14
Trial	1, 1314	6.08	.014	< .01
P1 accuracy (proportion)	1, 1314	16.65	< .001	.01
Predicted accuracy	1, 1314	0.47	.491	< .01
P2 accuracy (proportion)	1, 1314	5.83	.016	< .01
**Winner**	**1, 1314**	**0.38**	**.537**	**< .01**
Interaction model 1				
Difficulty x p1 accuracy	1, 1428	26.87	< .001	.02
Difficulty x p1 accuracy x winner	2, 1428	1.48	.227	< .01
Interaction model 2				
Difficulty x p2 accuracy	1, 1311	5.72	.017	< .01
**Difficulty x p2 accuracy x winner**	**2, 1311**	**9.01**	**< .001**	**.01**
Interaction model 4				
Helper memory x p2 accuracy	1, 1311	6.96	.008	.01
**Helper memory x p2 accuracy x winner**	**2, 1311**	**8.62**	**< .001**	**.01**

Note. Significant focal effects are bolded. Helper memory was coded as weak-memory = 0, strong-memory = 1.

Interaction model 2 (Difficulty x P2 Accuracy x Winner) Follow-up

Effects	*df*	*F*	*p*	η_p^2
P2 accuracy slope, p1 wins, 5-targets	1, 1311	3.85	.200	< .01
P2 accuracy slope, p1 wins, 10-targets	1, 1311	0.03	> .999	- < .01
P2 accuracy slope, p2 wins, 5-targets	**1, 1311**	6.11	.054	- < .01
**P2 accuracy slope, p2 wins,** 10-targets	**1, 1311**	**29.42**	**< .001**	**- .02**

Note. Significant winner effects are bolded. Bonferroni corrections are applied.

Interaction model 3 (P2 Accuracy x Helper Memory x Winner) Follow-up

Effects	*df*	*F*	*p*	η_p^2
P2 accuracy slope, p1 wins, weak-memory	1, 1311	0.32	> .999	< .01
P2 accuracy slope, p1 wins, strong-memory	1, 1311	3.74	.214	< .01
**P2 accuracy slope, p2 wins, weak-memory**	**1, 1311**	**29.64**	**< .001**	**.02**
**P2 accuracy slope, p2 wins, strong-memory**	**1, 1311**	**6.44**	**.045**	**< .01**

Note. Significant winner effects are bolded. Bonferroni corrections are applied.
